# Atrioventricular Synchronization for Detection of Atrial Fibrillation and Flutter in One to Twelve ECG Leads Using a Dense Neural Network Classifier

**DOI:** 10.3390/s22166071

**Published:** 2022-08-14

**Authors:** Irena Jekova, Ivaylo Christov, Vessela Krasteva

**Affiliations:** Institute of Biophysics and Biomedical Engineering, Bulgarian Academy of Sciences, Acad. G. Bonchev Str. Bl 105, 1113 Sofia, Bulgaria

**Keywords:** electrocardiogram diagnosis, arrhythmia, RR interval, PQ interval, PQ amplitude, artificial intelligence, dense neural networks, deep learning, SHAP value, biomedical signal processing, PhysioNet CinC Challenge 2021 database

## Abstract

This study investigates the use of atrioventricular (AV) synchronization as an important diagnostic criterion for atrial fibrillation and flutter (AF) using one to twelve ECG leads. Heart rate, lead-specific AV conduction time, and P-/f-wave amplitude were evaluated by three representative ECG metrics (mean value, standard deviation), namely RR-interval (RRi-mean, RRi-std), PQ-interval (PQi-mean, PQI-std), and PQ-amplitude (PQa-mean, PQa-std), in 71,545 standard 12-lead ECG records from the six largest PhysioNet CinC Challenge 2021 databases. Two rhythm classes were considered (AF, non-AF), randomly assigning records into training (70%), validation (20%), and test (10%) datasets. In a grid search of 19, 55, and 83 dense neural network (DenseNet) architectures and five independent training runs, we optimized models for one-lead, six-lead (chest or limb), and twelve-lead input features. Lead-set performance and SHapley Additive exPlanations (SHAP) input feature importance were evaluated on the test set. Optimal DenseNet architectures with the number of neurons in sequential [1st, 2nd, 3rd] hidden layers were assessed for sensitivity and specificity: DenseNet [16,16,0] with primary leads (I or II) had 87.9–88.3 and 90.5–91.5%; DenseNet [32,32,32] with six limb leads had 90.7 and 94.2%; DenseNet [32,32,4] with six chest leads had 92.1 and 93.2%; and DenseNet [128,8,8] with all 12 leads had 91.8 and 95.8%, indicating sensitivity and specificity values, respectively. Mean SHAP values on the entire test set highlighted the importance of RRi-mean (100%), RR-std (84%), and atrial synchronization (40–60%) for the PQa-mean (aVR, I), PQi-std (V2, aVF, II), and PQi-mean (aVL, aVR). Our focus on finding the strongest AV synchronization predictors of AF in 12-lead ECGs would lead to a comprehensive understanding of the decision-making process in advanced neural network classifiers. DenseNet self-learned to rely on a few ECG behavioral characteristics: first, characteristics usually associated with AF conduction such as rapid heart rate, enhanced heart rate variability, and large PQ-interval deviation in V2 and inferior leads (aVF, II); second, characteristics related to a typical P-wave pattern in sinus rhythm, which is best distinguished from AF by the earliest negative P-peak deflection of the right atrium in the lead (aVR) and late positive left atrial deflection in lateral leads (I, aVL). Our results on lead-selection and feature-selection practices for AF detection should be considered for one- to twelve-lead ECG signal processing settings, particularly those measuring heart rate, AV conduction times, and P-/f-wave amplitudes. Performances are limited to the AF diagnostic potential of these three metrics. SHAP value importance can be used in combination with a human expert’s ECG interpretation to change the focus from a broad observation of 12-lead ECG morphology to focusing on the few AV synchronization findings strongly predictive of AF or non-AF arrhythmias. Our results are representative of AV synchronization findings across a broad taxonomy of cardiac arrhythmias in large 12-lead ECG databases.

## 1. Introduction

Atrial fibrillation (AFIB) and atrial flutter (AFL) are the most common cardiac arrhythmias, being especially threatening for the geriatric population, with incidence increasing from 0.5% for people aged 40–50 years to 5–15% for people 80 years old [1,2]. Despite the different pathophysiology of AFIB and AFL, both are diseases associated with structural and electrical abnormalities of the atrium that increases the risk of stroke, heart failure, thromboembolism, and mortality; therefore, early diagnosis is vital [1,3,4,5].

**Figure 1:**
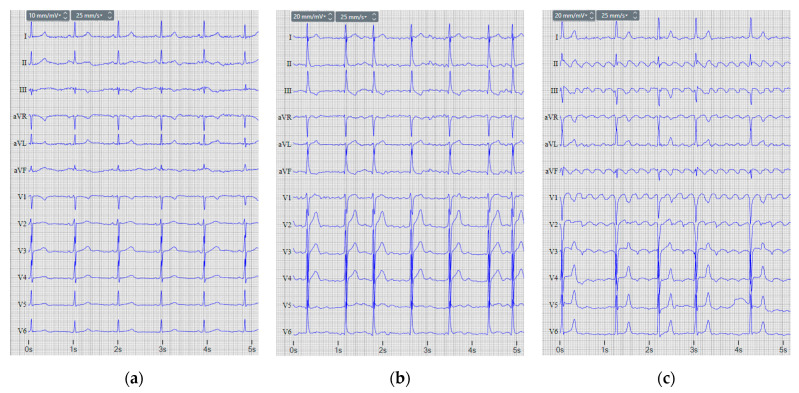
12-lead ECG records of: (**a**) normal sinus rhythm, (**b**) atrial fibrillation, and (**c**) atrial flutter.

During sinus rhythm, the depolarization of the cardiac muscle begins at the sinus node. In 12-lead ECGs, this is characterized by the presence of correctly oriented P-waves [6], which are positive in leads I, II, and aVF; negative in the lead aVR; biphasic (−/+), positive or negative in the lead aVL; and positive in all chest leads, except for V1 which may be biphasic (+/−), as illustrated in the example of Figure 1a. In contrast, AFIB and AFL are abnormal heart rhythms associated with irregular excitation of the atrial chambers; therefore, P-wave synchronization and morphology are completely distorted. Although AFIB and AFL both have increased atrial activity, their representation on the electrocardiogram (ECG) is not identical. AFIB is characterized by irregular atrial activity, discerned by high-frequency small-amplitude fibrillatory (f) waves instead of P-waves (Figure 1b). In general, f-waves trigger ventricular activity in a random manner, resulting in disturbed atrioventricular (AV) synchronization (PQ-intervals) and randomly fluctuating beat-to-beat intervals (RR-intervals) [5,7]. Therefore, AFIB detection relies on the identification of multiple f-waves [8,9] or the absence of P-waves [10,11]; estimation of RR-intervals in terms of median absolute deviation [12], irregularity [13], sample entropy [14], high-level features [15], and heart rate variability [16,17,18]; combined analysis of atrial and ventricular activities [19,20,21,22]; combined analysis of heart rate and variability of QT-intervals, QRS durations, or RR-intervals [23]; analysis of the cardiac cycle shape [24]; study of the ECG spectrogram [25]; involvement of extended feature sets describing the ECG in the time and frequency domain [26,27]; analysis of the raw ECG signal alone [28,29,30,31,32,33,34,35,36,37,38,39,40,41,42] and in combination with morphological features [43]; and f-wave spectra combined with RR intervals [44].

Typical AFL is observed with an inverted saw-tooth flutter (F) wave pattern in the inferior ECG leads II, III, and aVF; low amplitude biphasic F-waves in leads I and aVL; an upright F-wave in precordial lead V1; and an inverted F-wave in lead V6 (Figure 1c). In turn, atypical AFL is recognized with a less specific F-wave pattern in the 12-lead ECG, often with a sine wave pattern in inferior ECG leads [45]. Both AFL types exhibit regular atrial contractions that either provoke regular ventricular beats [46] or maintain patterns of irregularity (e.g., repeating short-short-long pattern) [47]. Therefore, AFL detection relies on a few ECG characteristics, mainly related to the analysis of P-waves, threshold crossing [8,48], or slices from the raw ECG signal [28,33].

Various machine learning classifiers have been applied to AFIB and AFL detection, based on their distinctive ECG features, by means of decision rules [22,48], random forest classifiers [26], cascaded support vector machines [26], Error-Correcting Output Codes [21], and shallow neural networks (NN) [27]. Recent developments of deep NN involve the direct analysis of raw ECG leads in combination with ECG features. We note the most popular techniques such as convolutional neural networks (CNN) [25,30,31,32,33,34,35,43,44], hybrid NN with CNN and Long-Short-Term Memory (LSTM) layers [15,39,40,41,42], CNN with enhanced Elman NN [28], or the LSTM autoencoder of cardiac cycles with a classifier involving a fully connected layer [24]. LSTM are the most common layers in recurrent neural networks (RNN), which manage sequential data of the ECG waveform. Examples of different RNN designs for cardiac arrhythmia classification are shown in [18,29,36,37,38]. 

According to the National Institute for Health and Care Excellence (NICE), AFIB diagnosis should be confirmed in a clinical environment with standard 12-lead ECG records [49]. Recently, P- and f-wave analysis in extended 15-lead ECG configurations have been shown to further improve AFIB detection accuracy [50,51]. On the other hand, short and asymptomatic AFIB episodes are difficult to identify in a clinical environment. Therefore, long-term ECG monitoring techniques, such as Holter ECG, loop recorders, or finger-based (Lead I) ECG devices that can trace a patient’s rhythm from a few minutes to several days are shown to be effective for detection of paroxysmal AFIB by reduced single-lead ECG analysis [7,52,53,54,55]. Due to differences in ECG lead availability for clinical and ambulatory diagnostics of AFIB, the recent PhysioNet Computing in Cardiology (CinC) Challenge 2021 set up the mandatory task of arrhythmia classification using different lead sets ranging from two to twelve ECG leads amongst the vast amount of 12-lead ECG recordings. This classification also included AFIB and AFL annotation labels [56]. 

Due to the black-box nature of NN, it is challenging to understand what has caused a certain output by a model, and for medical experts to be able to judge the validity of the diagnosis. In order to interpret the decision-making process in different machine learning classifiers, many studies have investigated the influence of input features on the classification output by means of the Gini index [57]; ‘weight’ method [57]; bagged decision tree ensemble [58]; Gain index [59]; minimum redundancy and maximum relevance approach [60,61]; and logistic regression, permutation testing, random forest, and SHapley Additive exPlanations (SHAP) [26]. Two recent studies on AFIB detection revealed the importance of the input feature map using two techniques for a functional understanding of the decision-making process in NN. The first study introduced a method that cumulatively estimates the weights of the activated neurons throughout the total path, from the input to the specific output neuron in a dense NN with 137 input ECG features, up to three dense (fully connected) hidden layers, and four fully connected output neurons responsible for AFIB prediction [27]. The second study calculated SHAP values for the input, using raw ECG samples of up to 12-leads over 20 s in a deep CNN architecture for classification of 26 cardiac abnormalities, including AFIB and AF [62].

This study aims to assess AV synchronization as an important diagnostic criterion for AFIB and AFL using one to twelve ECG leads. We focus on the delineation of just three representative ECG features, namely RR-interval, PQ-interval and PQ-amplitude, as an estimate of the heart rate, lead-specific AV conduction time, and P-/f-wave amplitude. We used an input feature map of a dense NN classifier to detect AFIB and AFL from a broad taxonomy of cardiac arrhythmias in large 12-lead ECG databases, based on estimations of beat-by-beat stability using mean values and standard deviations. By identifying the optimal design of various dense NN topologies using input from different sets of leads, we aim to give a comprehensive overview of different lead sets because of their varying availabilities in clinical settings. Furthermore, we are interested in evaluating the feature map interpretation by trained NN; therefore, the SHAP method was applied to highlight the most important 12-lead AV synchronization metrics used for AFIB and AFL detection. By broadly investigating AV synchronization features for their potential in lead-selection and feature-selection practices, we aimed to improve the clinical detection of AFIB and AFT. The real benefit would come from objectively weighing the importance of the input feature map, and changing the focus of the expert eye or automated interpretation algorithms to the heart rate dynamics and lead-specific PQ intervals that are highly predictive of AFIB and AFL.

The article has the following structure: Section 2 (“Materials and Methods” section) describes the ECG databases and study design, including the main steps of ECG pre-processing, feature extraction, design, and training of NN classifiers, as well as a description of the metrics used for performance evaluation and input feature importance estimation. Section 3 (“Results” section) shows the optimization process and justifies the selection of the best performing NN architectures, which used different input ECG feature maps from one, six, and twelve ECG leads. The strongest AF predictors are ranked with respect to their SHAP feature importance, giving a comprehensive analysis of the causes for true positives, true negatives, false positives, and false negatives, based on statistical distributions deduced from the test set and several examples. Section 4 (“Discussion” section) summarizes the results and compares our method with other classical and machine learning methods for AF arrhythmia detection, noting the disparities between studies with respect to databases and performance metrics. Section 5 (“Conclusions” section) highlights the clinical benefits and contributions of this study.

## 2. Materials and Methods

### 2.1. ECG Databases

We used the PhysioNet CinC Challenge 2021 databases [56,63], which are presently known as the largest freely available repository of standard 12-lead ECG records and consistent annotations for 30 clinical diagnoses of cardiac abnormalities (and/or a normal sinus rhythm), available on the Physionet website (https://physionet.org/content/challenge-2021/1.0.2/, last accessed on 21 June 2022). The scope of this study was included the specific diagnostic labels of AFIB and AFL arrhythmia; however, these were not separately annotated in some of the databases. Therefore, we consider both arrhythmias in a mixed class, namely AF (AFIB+AFL). All other records with diagnostic annotations different from either AFIB or AFL were considered to be in the non-AF class. The rate of AF vs. non-AF cases was about 8 vs. 92% from the total database of 71545 ECG records (Table 1). We maintained similar AF to non-AF proportions in random patient-wise allocation in three independent subsets: training (70%), validation (20%), and test (10%). 

Table 1 summarizes the entire number of short-term ECG records (duration 5–144 s) available in the six largest PhysioNet CinC Challenge 2021 databases, restricted by two exclusion criteria:

- ECG records that did not contain any of the annotation labels for the 30 clinical diagnoses defined for the Challenge.
- ECG records annotated as having low QRS voltages, poor R wave progression, or pacing rhythm.

### 2.2. Study Design

**Figure 2:**
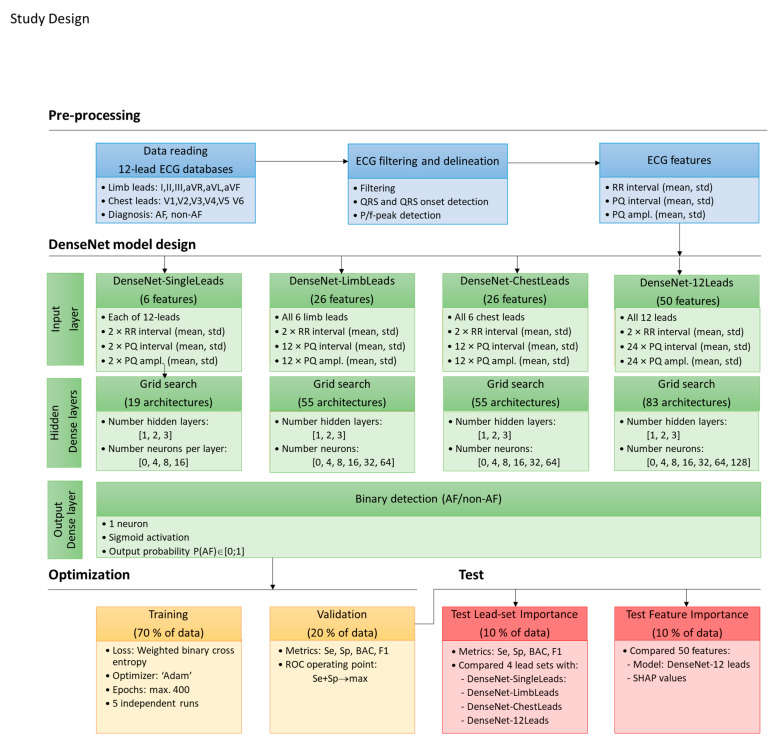
Study design for binary AF/non-AF detection with dense NN classifier (DenseNet), using RR-interval, PQ-interval, and PQ-amplitude input features (mean value, standard deviation) from different lead sets in 12-lead ECG. Grid search architectural design and optimization was applied to derive the best DenseNet models, which were tested to study the importance of lead-set and input features.

Figure 2 presents the study design for a binary AF/non-AF rhythm classification, which consisted of four major parts:

1. Pre-processing: Analysis of full-length 12-lead ECG records with a focus on QRS, QRS onset, and P-/f-peak detection, as well as measurement of RR-intervals, lead-specific PQ-intervals, and PQ-amplitudes.
2. DenseNet model design: Grid-search architectural design of dense NN classifiers with input features from different lead sets, including single lead, six limb leads, six chest leads, and all twelve leads.
3. Optimization: Training and validation process for selection of the best models with maximal performance.
4. Test: Performance evaluation on the independent test set, which derived conclusions on the importance of lead-set and input features.

The subsequent sections describe each part of the study design.

### 2.3. Pre-Processing

#### 2.3.1. Data Reading

The input data were read with the open-source Python example code for the PhysioNet/Computing in Cardiology Challenge 2021 [64], available at the link: https://github.com/physionetchallenges/python-classifier-2021, last accessed on 21 June 2022. The standard 12-lead ECG raw signals of limb leads (I, II, III, aVR, aVL, and aVF) and chest leads (V1–V6) were loaded as binary MATLAB v4 files, resampled to a common frequency of 500 Hz. The annotations were read as plain text files in WFDB header format [65] for the recording, patient attributes, and the diagnosis, originally stored as SNOMED-CT codes with validated annotations, as described at the link: https://github.com/physionetchallenges/evaluation-2021/blob/main/dx_mapping_scored.csv, last accessed on 21 June 2022. Considering that one ECG recording could have one or more diagnostic labels for cardiac abnormalities, the presence of one of the codes “164889003” (AF) or “164890007” (AFL) assigned the record to the AF class. Otherwise, it was assigned to the non-AF class. According to the exclusion criteria in Section 2.1, all records without a code or with at least one of the following codes were excluded: “365413008” (poor R wave progression), “251146004” (low QRS voltages), or “10370003” (pacing rhythm). 

#### 2.3.2. ECG Filtering and Delineation

The noise components in each ECG lead were suppressed by three filtering procedures, which were originally designed to preserve low- and high-frequency ECG waves, including: (i) the subtraction procedure for power-line interference cancellation with dynamic adjustment to linear and non-linear ECG segments [66]; (ii) high-pass recursive filter with a cutoff frequency of 0.64 Hz for removal of the baseline drift [67]; (iii) low-pass Savitzky-Golay filter for suppression of electromyographic (EMG) noise with dynamic adaptation of the cutoff frequency to about 14 Hz (low-power P, T-waves, PQ, ST, and TP-segments), 20–30 Hz (high-power P and T-waves), and >100 Hz (QRS complexes), to best keep the frequency spectra of specific ECG waves [68].

**Figure 3:**
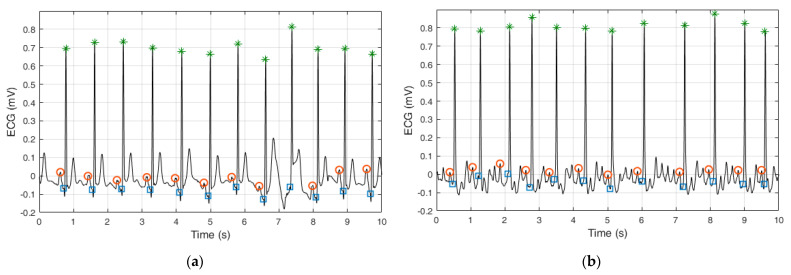
ECG delineation applied in this study, including detection of R-waves (green stars), QRS onsets (blue squares), and preceding P-/f-wave peaks (red circles), illustrated for examples of (**a**) non-AF rhythm and (**b**) AF rhythm.

This study applies simple ECG delineation methods of three characteristic points within each ECG cycle, namely the R-wave (by QRS detection), Q-wave (by QRS onset detection), and P-/f-peak (by detection of one deflection wave preceding the Q-wave). An illustration of these three characteristic points is presented in Figure 3, with examples showing normal sinus rhythm and AF rhythm.

Our QRS detector used the real-time algorithm of Christov [69], which identifies significant peaks of spatial velocity (absolute value of the first derivative of one or more ECG leads) using three adaptive thresholds for the QRS amplitude, slew rate, and high-frequency noise. The QRS detector operates with any number of ECG leads, self-synchronizes to QRS slopes, and adapts to beat-to-beat intervals. Its efficiency was high enough (about 99.7% [69]) for reliable QRS detection in this study.

The QRS onset (Q) is found in the first isoelectric segment before the QRS fiducial point (R-wave) applying a low-slope criteria within 20 ms [70,71]:

(1) $$|{\mathrm{ECG}}_{t}-{\mathrm{ECG}}_{t-20\mathrm{ms}}{|}_{t=0}^{20\mathrm{ms}}\leq {\mathrm{Thr}}_{Q}\;\mathrm{and}\;\left|{\mathrm{ECG}}_{t}-{\mathrm{ECG}}_{t-20\mathrm{ms}}\right|\leq 4{\mathrm{Thr}}_{Q}\;\mathrm{and}\;\left|{\mathrm{ECG}}_{t}-{\mathrm{ECG}}_{t-10\mathrm{ms}}\right|\leq 3{\mathrm{Thr}}_{Q}\;\mathrm{and}\;\left|{\mathrm{ECG}}_{t-10\mathrm{ms}}-{\mathrm{ECG}}_{t-20\mathrm{ms}}\right|\leq 3{\mathrm{Thr}}_{Q}$$

where ECG_t_ denotes the ECG sample at time t ∈[R−120 ms;R]; Thr_Q_ = 20 μV is the default value of the threshold slope for Q-wave detection, which is incremented by 1 μV until all conditions in (1) are satisfied.

The peak of the P-wave (non-AF) or f-wave (AF) is searched within a physiologically reasonable interval before the Q-wave, t∈[Q−300 ms;Q−40 ms], applying criteria for a high-slope deflection wave within 80 ms; the relevance of this was proven in a competitive study of the PhysioNet/Computing in Cardiology Challenge 2017 [72]. The specific conditions are as follows:

(2) $$\left|{\mathrm{ECG}}_{t}-{\mathrm{ECG}}_{t-40\mathrm{ms}}\right|>4{\mathrm{Thr}}_{P}\;\mathrm{and}\;\left|{\mathrm{ECG}}_{t}-{\mathrm{ECG}}_{t+40\mathrm{ms}}\right|>{\mathrm{Thr}}_{P}\;\mathrm{and}\;\left|{\mathrm{ECG}}_{t}-{\mathrm{ECG}}_{t-20\mathrm{ms}}\right|>{\mathrm{Thr}}_{P}\;\mathrm{and}\;\left|\mathrm{ECG}-{\mathrm{ECG}}_{t+20\mathrm{ms}}\right|>{\mathrm{Thr}}_{P}/2$$

where Thr_p_ is the threshold slope for P-/f-peak detection with an empirically defined value of 3 μV. If none of the conditions (2) are satisfied for any t in the defined search interval, then no P-/f-peak was detected before the Q-wave. This scenario is illustrated in Figure 3a for the QRS complex between the 7th and 8th second, where a supposed artifact altered the P-peak. We note that all other beats in Figure 3 present sufficiently well-detected series of the three characteristic points (R-wave, Q-wave, and P-/f-peak).

#### 2.3.3. ECG Features

The key focus for simple AF detection are three diagnostic ECG features, representative of the ventricular rate (RR-interval: RRi), AV synchronization time (PQ-interval: PQi), and P-/f-wave amplitude (PQ-amplitude: PQa), statistically evaluated as mean values and standard deviations (std) over the total available ECG record length (5-144s). The list of computed features is as follows:

- RRi-mean and RRi-std (two global features) are computed as the mean and std values of the distances between consecutive R-wave fiducial points [73], detected in this study in reference lead I. If QRS detection is assumed correct, its application to any other lead is expected to give the same estimation of the heart rate; therefore, RRi-mean and RRi-std are considered global features.
- PQi-mean and PQi-std (two lead-specific features) are computed as the mean and std values of the time distances from P-/f-peaks to subsequent Q-waves in each of the 12 ECG leads. We note that the computed PQi-mean value differs from the standard definition of the PQ interval between the beginning of the P-wave and the beginning of the Q-wave [6]. The reason for this is that the embedded automatic delineation algorithms (Equation (2)) detects the most characteristic P-/f-peak more reliably than the wandering onset of the P-/f-wave. Although the computed PQ interval would be slightly shorter than the defined normal ranges [6], we consider its reliable measurement an important requirement for the proper investigation of the AF predictive potential of this feature.
- PQa-mean and PQa-std (two lead-specific features) were computed as the mean and std values of the amplitude differences between P-/f-peaks and subsequent Q-waves in each of the 12 ECG leads. We consider the PQa-mean value to represent the largest deflections of atrial electrical activity discernible before QRS onset and is therefore representative of the P-/f-wave amplitude. We believe that the detection of the Q-wave reference level is more reliable than searching for the P-wave onset, as defined in the standard P-wave amplitude measurement [6].

We note that if a P-/f-peak is not detected before a Q-wave, then the respective measurements are not included in the statistical computations of PQi-mean, PQi-std, PQa-mean, and PQa-std.

The computation of four lead-specific features (PQi-mean, PQi-std, PQa-mean, and PQa-std) for each of the 12 ECG leads gives the opportunity to apply the concept of varying dimensions in electrocardiography. According to this concept, AF rhythm diagnosis could be made possible according to the lead availability in an arbitrary clinical setting, e.g., one-lead (using any of 12 leads by means of 6 features: 2RRi + 2(PQi = PQa)), six-lead (using limb or chest leads by means of 26 features: 2RRi + 6 × 2(PQi + PQa)), or twelve-lead (using all 12 leads by means of 50 features: 2RRi + 12 × 2(PQi + PQa)).

### 2.4. DenseNet Model Design

**Figure 4:**
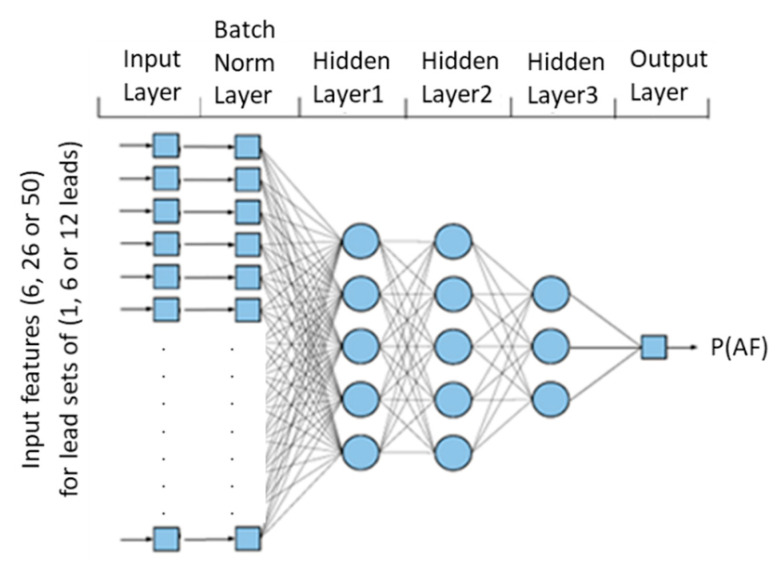
Configurable DenseNet architecture used in this study.

The study design in Figure 2 follows the concept of varying dimensions in electrocardiography for binary AF/non-AF rhythm classification, which is related to the optimal design of various dense NN topologies using input with different sets of leads, namely DenseNet-SingleLeads for one lead, DenseNet-LimbLeads for six limb leads, DenseNet-ChestLeads for six chest leads, and DenseNet-12Leads for all standard 12 leads. All DenseNet models have a common architecture, which is schematically drawn in Figure 4, and can be configured as follows:

- Input layer: the number of nodes is equal to the number of features, as defined in Section 2.3.3., and computed by the formula: (2 + number of leads × 4), i.e., 6 features (DenseNet-SingleLeads), 26 features (DenseNet-LimbLeads, DenseNet-ChestLeads), and 50 features (DenseNet-12Leads).
- Batch normalization (BN) layer: a regularization technique that is known to accelerate training [74]. In our model, BN is applied for standardization of the input feature (x) by removing the mean and scaling to unit variance x_BN_ = (x – mean)/(std) for each mini-batch. BN transform layer BN_γ,β_ ≡ γx_BN_ + β computes two trainable parameters (γ, β) for each input feature x.
- Hidden dense layers: a sequence of hidden dense layers for feature fusion and multilevel abstraction of feature maps [75]. One dense layer neuron processes the information of the feature vector x, according to the transform:

(3)  z=max(∑Wx+b,0)

where W and b are, respectively, the kernel weights matrix and the bias of the neuron to which a rectified linear unit activation function is applied.

The following setting of the hidden dense layers are considered reasonable to configure the network depth and width:

- One to three hidden layers can be allocated.
- The number of neurons in one dense layer cannot be larger than the number of neurons in the previous dense layer, limiting DenseNet to a shrinking architecture.
- The number of neurons in a hidden dense layer can be any in the list:
  ✓ [0, 4, 8, 16] for DenseNet-SingleLeads;
  ✓ [0, 4, 8, 16, 32, 64] for DenseNet-LimbLeads or DenseNet-ChestLeads;
  ✓ [0, 4, 8, 16, 32, 64, 128] for DenseNet-12Leads.

- Output layer: a dense layer with one neuron and sigmoid activation function, giving the probability of the feature vector x belonging to the AF class in the range [0; 1]:

(4) $$P(x\;\in \mathrm{AF})=\sigma (z)=\frac{1}{1+\exp\left(-z\right)}\;$$

Although not depicted in the general network topology in Figure 4, a drop-out regularization layer is applied after each hidden dense layer to avoid over-fitting and improve generalization during training. The drop-out rate of α = 0.3 was adopted as it was a common setting effectively applied in several studies [27,76].

### 2.5. DenseNet Model Training

A random uniform kernel weights initializer, DenseNet model fit by ‘Adam’ optimizer with a default learning rate of 0.001, and exponential decay rates of β1 = 0.9 (first moment) and β2 = 0.999 (second moment) were set for the training phase. ‘Adam’ optimizes the parameters θ of the network, in order to minimize loss:

(5) $$\theta =\underset{\theta }{\mathrm{argmin}}\frac{1}{N}\overset{N}{\underset{n}{\sum }}\mathrm{Loss}(x_{n},\theta )$$

where x_n_ is a feature vector of sample n in the training dataset (or batch size) with a number of N samples, and Loss is computed as a weighted binary cross entropy due to the notable imbalance between AF and non-AF classes:

(6) $$\mathrm{Loss}=-\frac{1}{N}\overset{N}{\underset{n}{\sum }}\delta_{n}w_{\mathrm{AF}}\log\left(P\left(x_{n}\in \mathrm{AF}\right)\right)+\left(1-\delta_{n}\right)w_{\mathrm{non}-\mathrm{AF}}\log\left(1-P\left(x_{n}\in \mathrm{AF}\right)\right)$$

where:

- δ_n_ is a binary indicator function, which is equal to 1 if the training sample x_n_ belongs of the AF class, otherwise δ_n_ = 0;
- w_AF_ and w_non − AF_ are the weights for AF and non-AF classes, respecting the condition w_AF_ + w_non – AF_ = 1. Considering the proportion of about 8% AF to 92% non-AF in the training database (Table 1), the class weights were configured to give a penalty to the larger class, computing was a reciprocal of the class prevalence, i.e., w_AF_ = 0.92, w_non – AF_ = 0.08.

The model with minimal loss over the validation set, trained for a maximum of 400 epochs, was stored in an HDF5 file. Early stopping was activated if loss was not improved for >10 epochs. The DenseNet models were implemented in Python using Keras with Tensorflow backend. The training was run on workstation PERSY Stinger with Intel CPU Xeon Silver 4214R at 2.4 GHz (2 processors), 96 GB RAM, NVIDIA RTX A5000-24GB GPU.

### 2.6. Performance Evaluation

The performance of trained DenseNet models was estimated by benchmark metrics of sensitivity (Se), specificity (Sp), balanced accuracy (BAC), and F1 score:

(7) $$\mathrm{Se}=100\frac{\mathrm{TP}}{\mathrm{TP}+\mathrm{FN}}\;,\;\left[\%\right]$$

(8) $$\mathrm{Sp}=100\frac{\mathrm{TN}}{\mathrm{TN}+\mathrm{FP}},\;\left[\%\right]$$

(9) $$\mathrm{BAC}=\frac{\mathrm{Se}+\mathrm{Sp}}{2},\;\left[\%\right]$$

(10) $$F1=\frac{2\mathrm{TP}}{2\mathrm{TP}+\mathrm{FN}+\mathrm{FP}}$$

where TP and FN are the true positive and false negative detections for the AF class, and TN and FP are the true negative and false positive detections for the non-AF class.

We note that BAC and F1 scores are common performance metrics for classifying imbalanced data. Furthermore, BAC→max is a representative point in the receiver operating characteristic (ROC) curve, giving the highest averaged performance of both classes and corresponding to the highest pair (Se,Sp)→max [76]. Therefore, the optimal choice of the output probability threshold for detection of AF ($x\equiv \mathrm{AF}\;\mathrm{while}\;P\left(x\;\in \mathrm{AF}\right)>P_{\mathrm{thr}})$ is set at the ROC operating point $\left(P_{\mathrm{thr}}\in {\mathrm{BAC}}_{validation}\to \max\right),$ where ROC is computed for the validation dataset. The top-ranked models by BAC*_validation_* with their respective P_thr_ value were selected for further independent evaluation on test set performance (Se, Sp, BAC, and F1 score).

### 2.7. Feature Importance

Estimation of input feature importance is an essential to understanding the decision-making process in DenseNet hidden layers. We were interested to interpret the feature map learned by the top-ranked DenseNet-12Leads model, which used the full-set of 50 input features for AF/non-AF classification. This interpretation was expected to highlight the most reliable ECG characteristics among heart rate and its variability, AV synchronization, and P-/f-wave amplitude and its stability in each of the 12 ECG leads.

We implemented SHAP [77], as one of the famous and powerful methods for explanation of individual predictions of various machine learning classifiers, based on coalition game theory [78]. SHAP calculates the local feature importance for every observation as the average marginal contribution of a feature across all possible combinations of features (i.e., all possible coalitions):

(11) $${\mathrm{SHAP}\;\mathrm{value}}_{\mathrm{Fi}}=\underset{S\subseteq M-i}{\sum }\frac{\left|S\right|!\left(\left|M\right|-\left|S\right|-1\right)!}{\left|M\right|!}\left[f\left(S\cup \mathrm{Fi}\right)-f\left(S\right)\right]$$

- SHAP value_Fi_ is computed for the feature Fi, where i=1,2,…50 is the index of the input features of the DenseNet-12Leads model;
- M is the full set of features;
- S refers to a subset of features, which does not include the feature Fi;
- S ∪ Fi is a subset, which includes the features in S together with the feature Fi;
- S ⊆ M − i are all sets S, which are subsets of M and do not contain the feature Fi.

A SHAP value_Fi_ could be positive or negative, depending on the estimated contribution of the feature Fi for detection, either of the positive class (AF) or negative class (non-AF), respectively. The larger the absolute SHAP value_Fi_, the greater is the contribution of the feature Fi to the DenseNet output probability in Equation (4). However, the SHAP value in Equation (11) is only interpretable in the context of a specific ECG record. A global estimation of the importance of feature Fi for all N records in the test set can be calculated as an average of the absolute SHAP values in individual records:

(12) $$\mathrm{SHAP}\;{\mathrm{global}}_{Fi}=\frac{\sum_{n=1}^{N}\left|\mathrm{SHAP}\;{\mathrm{value}}_{Fi}\left(n\right)\right|}{N}$$

## 3. Results

### 3.1. DenseNet Model Optimization

**Figure 5:**
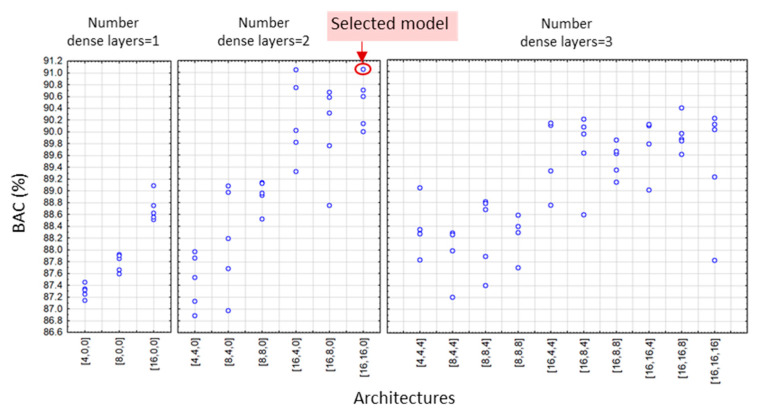
Validation BAC for AF detection of 95 trained DenseNet-SingleLeads models by grid search, including 5 runs of 3 models with 1 hidden layer, 6 models with 2 hidden layers, and 10 models with 3 hidden layers. The architecture on the x-axis denotes the number of neurons in the [1st, 2nd, 3rd] hidden dense layer. The red circle highlights high-ranked BAC = 91.1% of the selected optimal architecture DenseNet-SingleLeads [16,16,0].

**Figure 6:**
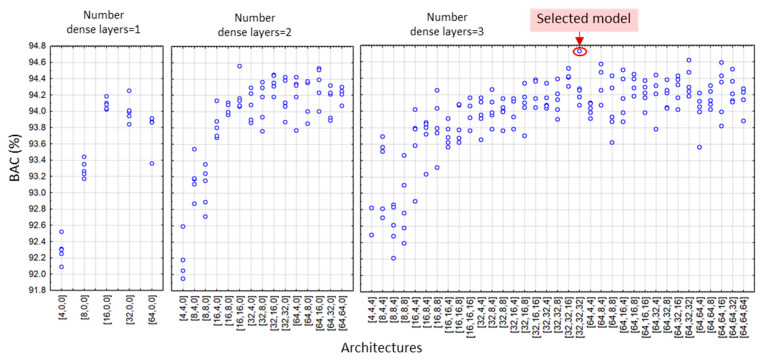
Validation BAC for AF detection of 275 trained DenseNet-LimbLeads models by grid search, including 5 runs of 5 models with 1 hidden layer, 15 models with 2 hidden layers, and 35 models with 3 hidden layers. The architecture on the x-axis denotes the number of neurons in the [1st, 2nd, 3rd] hidden dense layer. The red circle highlights high-ranked BAC = 94.7% of the selected optimal architecture DenseNet-LimbLeads [32,32,32].

**Figure 7:**
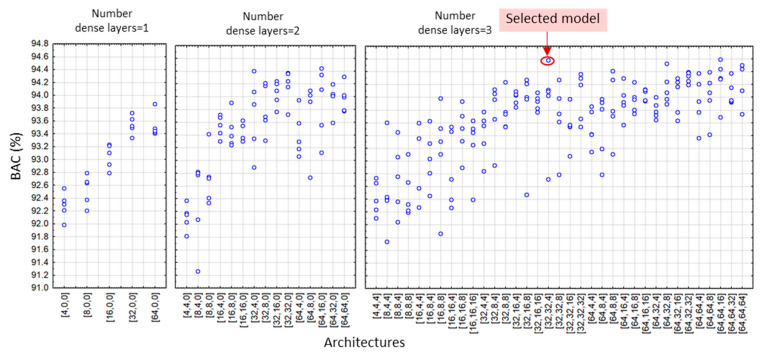
Validation BAC for AF detection of 275 trained DenseNet-ChestLeads models by grid search, including 5 runs of 5 models with 1 hidden layer, 15 models with 2 hidden layers, and 35 models with 3 hidden layers. The architecture on the x-axis denotes the number of neurons in the [1st, 2nd, 3rd] hidden dense layer. The red circle highlights high-ranked BAC = 94.6% of the selected optimal architecture DenseNet-ChestLeads [32,32,4].

**Figure 8:**
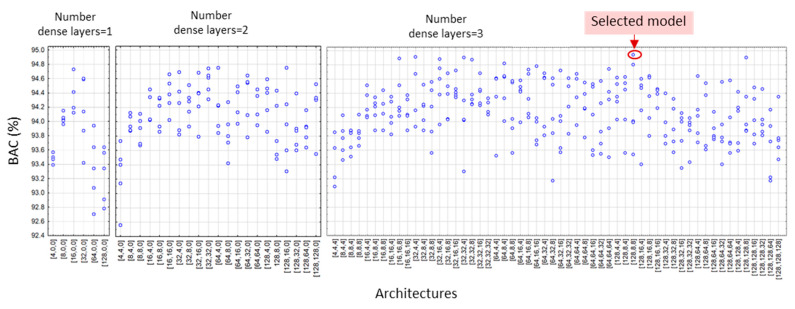
Validation BAC for AF detection of 415 trained DenseNet-LimbLeads models by grid search, including 5 runs of 6 models with 1 hidden layer, 21 models with 2 hidden layers, and 56 models with 3 hidden layers. The architecture on the x-axis denotes the number of neurons in the [1st, 2nd, 3rd] hidden dense layer. The red circle highlights high-ranked BAC = 94.9% of the selected optimal architecture DenseNet-12Leads [128,8,8].

According to the DenseNet model design settings in Section 2.4, we applied a grid search of all feasible combinations of the depths and widths of hidden dense layers, yielding a total number of 19 (DenseNet-SingleLeads), 55 (DenseNet-LimbLeads), 55 (DenseNet-ChestLeads), and 83 architectures (DenseNet-12Leads). Each architecture was trained and validated with five independent runs, resulting in a total number of 95 (DenseNet-SingleLeads), 275 (DenseNet-LimbLeads), 275 (DenseNet-ChestLeads), and 415 trained models (DenseNet-12Leads). The validation BAC of all DenseNet runs is illustrated in Figure 5, Figure 6, Figure 7 and Figure 8. Analysis of each lead configuration is further presented in the context of the BAC range for all runs and selection of the optimal DenseNet architecture, denoted by the number of neurons in sequential hidden layers [1st, 2nd, 3rd]:

- DenseNet-SingleLeads models with six input features from a single lead (Figure 5) presented validation BAC in the range 86.8–91.1%, reported as an average value for all 12 ECG leads where each lead was evaluated as an independent input. The best performance was observed for all architectures with two hidden dense layers with 16 neurons in the first layer. Our choice for the optimal model with high-ranked BAC = 91.1% was the DenseNet-SingleLeads [16,16,0].
- DenseNet-LimbLeads models with 26 input features from limb leads (Figure 6) presented validation BAC in the range 92–94.7%. Generally, the best performances (>94.2%) were observed for two and three hidden layer architectures with ≥32 neurons in the first layer. Our choice for the optimal model with high-ranked BAC = 94.7% was DenseNet-LimbLeads [32,32,32].
- DenseNet-ChestLeads models with 26 input features from chest leads (Figure 7) presented validation BAC in the range 91.8–94.6%. Generally, the best performances (>93.8%) were observed for two and three hidden layer architectures with ≥32 neurons in the first layer. Our choice for the optimal model with high-ranked BAC = 94.6% was DenseNet-ChestLeads [32,32,4].
- DenseNet-12Leads models with 50 input features from all 12 leads (Figure 8) presented validation BAC in the range 92.6–94.9%. The best performances (>94.8%) were observed for architectures with three hidden dense layers and ≥16 neurons in the first layer. Our choice of the optimal model with high-ranked BAC = 94.9% was DenseNet-12Leads [128,8,8].

### 3.2. Test Lead-Set Performance

Table 2 presents validation and test performance of the four selected optimal architectures in Figure 5, Figure 6, Figure 7 and Figure 8. Here, the single-lead model (DenseNet-SingleLeads [16,16,0]) is tested with 6 lead-specific features for each of 12 leads. Its summary performance on the total of lead-specific features is also presented. It is compared with the validation and test performances of limb, chest, and twelve-lead models with larger numbers of 26 and 50 features. The results show that the test performance was slightly inferior to that of the validation because the validation dataset was used during the training process of each model. We measure validation to test BAC drop of about 3% points for the single lead model (91.1 vs. 88.0%), 2% points for the limb lead (94.7 vs. 92.4%) or chest lead model (94.6 vs. 92.7%), and 1% points (93.8 vs. 94.9%) for the 12-lead model. These results suggest that a more accurate and a more robust AF diagnosis can be made by increasing the number of analyzed ECG leads.

**Figure 9:**
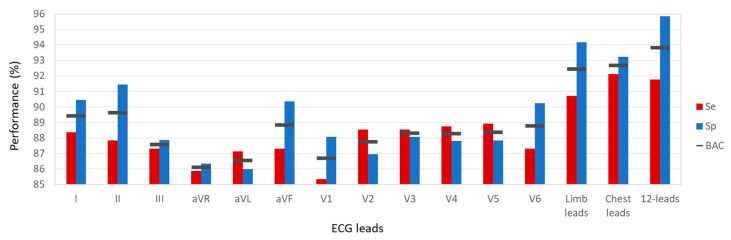
Test performance (Se, Sp, BAC) for binary detection of AF/non-AF rhythm by optimal models: DenseNet-SingleLeads [16,16,0] applied for individual leads (I, II, III, aVR, aVL, aVF, and V1-V6), DenseNet-LimbLeads [32,32,32] for limb leads, DenseNet-ChestLeads [32,32,4] for chest leads, DenseNet-12Leads [128,8,8] for the full set of 12 ECG leads.

Figure 9 presents the test performance of DenseNet-SingleLeads [16,16,0] when the model was evaluated for single leads (I, II, III, aVR, aVL, aVF, and V1-V6), giving an overview of the lead-specific importance of AF detection. The most efficient are the primary ECG leads (I, II), which achieve the highest single-lead BAC = 89.5% (Se = 87.9–88.3%, Sp = 90.5–91.5%). The third top-ranked lead was aVF, presenting an approximate 0.5% point performance drop compared with primary leads I and II. Chest leads V2–V5 had limited Sp < 88%, whereas V1 and V6 had limited Se < 87.5%. The use of multi-lead sets of peripheral or chest leads improved AF detection performance compared with the most powerful single lead, i.e., all peripheral leads improved up to 2.9% points more than reference lead II (Se = 87.9 vs. 90.8%, Sp = 91.5 vs. 94.1%); the improvement for all chest leads ranged from 3 to 5% points compared with reference lead V5 (Se = 89 vs. 92%, Sp = 87.8 vs. 93.1%). The use of all 12 ECG leads for AF diagnosis did not further improve Se. However, 12-leads did considerably improve Sp = 95.8%, which was 2 and 3% points larger compared with the limb or chest lead set, respectively. The latter suggests that the multi-lead view of the P-wave pattern in both frontal and horizontal planes is important for detection of the variety of rhythm and conduction disturbances in the non-AF class.

A detailed analysis of the test performance achieved with the complete feature set of the 12-lead ECG model (DenseNet-12Leads [128,8,8]) across six test datasets is presented in Table 3. We note a relatively large span for the Se (66.7 to 98.0%), Sp (88.4 to 97.1%), and F1 score (0.444 to 0.854), where the worst Se and F1-score were seen in the dataset with a limited number of AF (Georgia 12-Lead ECG Challenge database), whereas the worst Sp was seen in the CPSC2018 training set. The disparity in performance that one method could present on different test datasets was an indication of the inconsistency of the datasets with respect to their rhythm content and AF annotations. Nevertheless, reporting on multiple databases allows for greater generalizability of our results.

### 3.3. Test Feature Importance

This section describes our analysis of the 50 features of AV synchronization that were studied for their importance to AF/non-AF detection, evaluated by the test set. This provides a comprehensive overview of the decision-making process of the optimal DenseNet model, which is trained to most effectively combine information from all 12 ECG leads (DenseNet-12Leads [128,8,8]).

**Figure 10:**
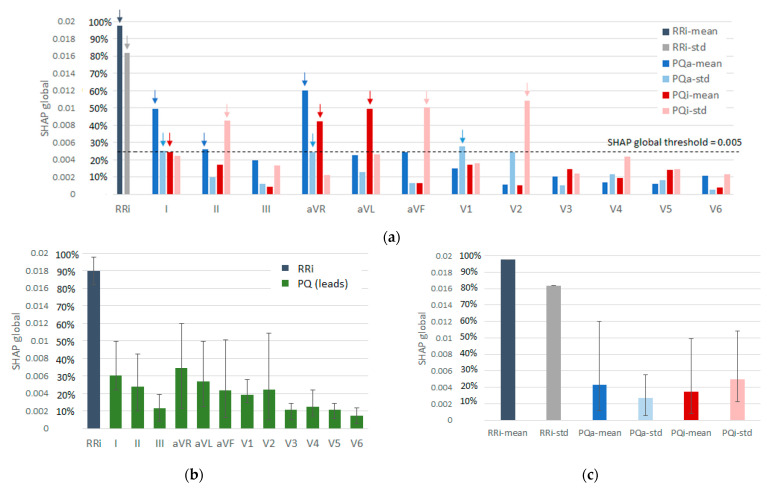
Importance of 50 input features for AF/non-AF detection by DenseNet-12Leads model, estimated by the SHAP global metric on the test set: (**a**) ranking of all 50 features and highlighting the top AF predictors with a SHAP global exceeding a threshold (vertical arrows); (**b**) ranking of 12 leads by summary of their PQ features as mean values (bars) and min–max ranges (whiskers); and (**c**) ranking of PQ features by their values in all 12 leads, presented as mean values (bars) and min–max ranges (whiskers). In all plots, RRi features were used as a reference with 100% for maximal SHAP at RRi-mean.

The SHAP global metric, computed as the mean SHAP value of the entire test set according to Equation (12), was used to scale feature importance along the y-axis in Figure 10. Figure 10a shows detailed information on the importance of each of the 50 features. This graph distinguishes the most important AF predictors with a SHAP global metric above a threshold, e.g., >0.005 set at about 25% of maximum importance. We highlight 14 important AF predictors with SHAP global (% of maximal importance) as follows: RRi-mean = 0.0196 (100%), RRi-std = 0.0164 (84%), PQa-mean (aVR) = 0.0120 (61%), PQi-std (V2) = 0.0108 (55%), PQi-std (aVF) = 0.0101 (52%), PQa-mean (I) = 0.0099 (51%), PQi-mean (aVL) = 0.00989 (50%), PQi-std (II) = 0.0085 (43%), PQi-mean (aVR) = 0.00847 (43%), PQa-std(V1) = 0.00556 (28%), PQa-mean (II) = 0.00518 (26%), PQa-std (I) = 0.0050 (26%), PQa-std (aVR) = 0.0050 (26%), and PQi-mean (I) = 0.0050 (26%). A comprehensive interpretation of this exhaustive list of features revealed that RR-intervals were the most valuable metric for AF detection, with a relative importance in the range of 84–100%. Furthermore, the importance of the other PQ metrics could be summarized in view of:

- Lead-set importance (Figure 10b):
  - Lead aVR is the most important lead due to three distinguished features: PQa-mean (61%), PQi-mean (43%), and PQa-std (26%).
  - Lead I is the second most important lead due to the same three features as aVR: PQa-mean (51%), PQi-mean (26%), and PQa-std (26%).
  - Lead aVL is ranked third for one feature: PQi-mean (50%).
  - Leads V2, aVF, and II have one highlighted feature: PQi-std (43-55%).
  - Lead V1 presents only one faintly distinguishable feature: PQa-std (28%).
  - Leads III, V3, V4, V5, and V6 do not present any important features.
- Feature importance (Figure 10c):
  - PQi-std is the most important feature mostly due to its high SHAP global metric in leads V2 (55%), aVF (52%), and II (43%).
  - PQa-mean is the second most important feature, distinguished in leads aVR (61%), I (51%), and II (26%).
  - PQi-mean is the third-ranked feature, distinguished in leads aVL (50%), aVR (43%), and I (26%).
  - PQa-std is the least important feature, weakly emphasized in leads V1 (28%), I (26%), and aVR (26%).

One should interpret the feature importance results considering the coalition game theory used in SHAP, which highlights the unique contribution of a feature to an output. This may underestimate a PQ feature measured in a set of correlated leads or features.

**Figure 11:**
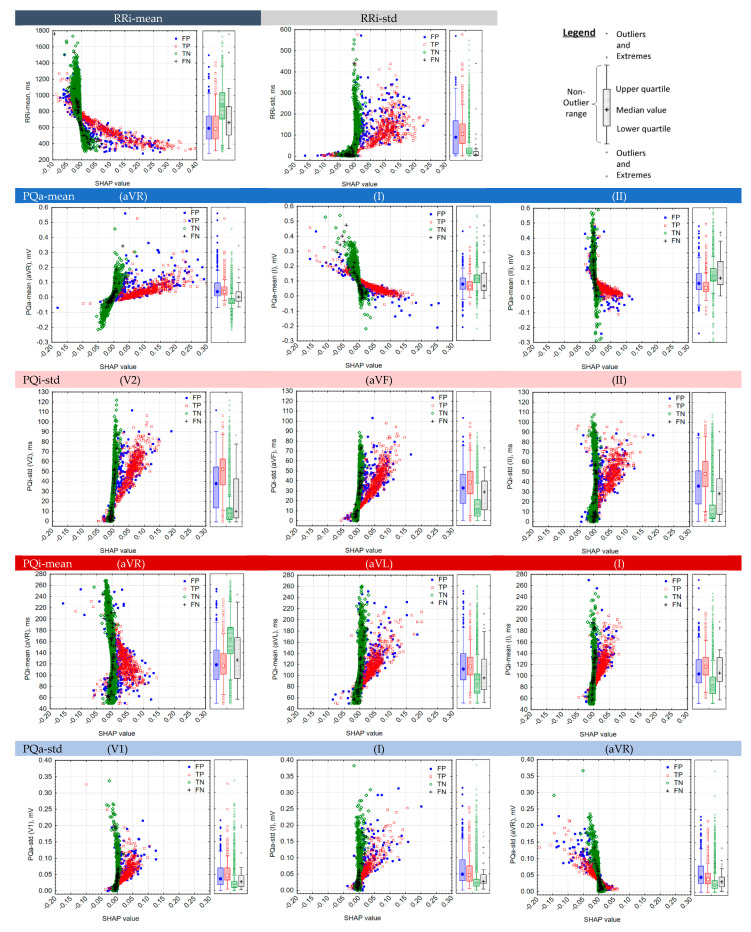
Statistical analysis of the feature values of 14 strongest AF predictors in 12-lead ECG, categorized according to FP, TP, TN, and FN detections by DenseNet-12Leads [128,8,8] in the test dataset. The statistical distributions are presented as box plots of the feature value (median value, interquartile range, non-outlier range, outliers, and extremes) and scatter plots of the feature value vs. SHAP values underlying its importance.

An important question was about the cause of the errors (FP and FN) in NN-driven AF detection. In Figure 11, we address this question in a statistical overview of the test set. Particularly, the feature values of the 14 strongest AF predictors in 12-lead ECG (highlighted in Figure 10a) are statistically evaluated in four groups: FN, TP, TN, and FP. These groups represent the output of the DenseNet-12Leads [128,8,8] model against the reference test set annotations (AF/non-AF). Figure 11 depicts two types of statistical distributions:

- Scatterplots (case study): feature values are scored in respect to their case feature importance (SHAP value).
- Box plots (group statistics): feature values are observed as global distributions (median value, interquartile range, non-outlier range, outliers, and extremes).

Valuable observations for the most important features can be deduced from Figure 11:

- RRi-mean: There is a trend showing that the shorter the RRi-mean, the greater the SHAP value for the importance of this parameter for TP (red dots), which can be explained by the usual rapid heart rates in AF. FP cases are also rapid rhythms, with an RRi-mean box plot distribution quite similar to TP (median RRi-mean = 600 ms, median heart rate HR = 100 bpm). Instead, the green dots present larger negative importance for TN detection for longer RRi-means (median RRi-mean = 880 ms, median HR = 68 bpm). The distribution of FN errors shows slightly decelerated AF rhythms (median RRi-mean = 650 ms, median HR = 92 bpm).
- RRi-std: A clearly visible trend shows that a greater RRi-std is directly proportional to greater SHAP importance for TP (red dots), associated with an increased heart rate variability in irregular ventricular activation during AF. FP cases also present increased RR-variability (median RRi-std = 100 ms). On the other hand, TN and FP cases are scored with reduced RR variability (median RRi-std < 20 ms).
- PQa-mean (aVR): The typically negative P-wave amplitude in the lead aVR of sinus rhythms (median PQa-mean = −0.025 mV) is highlighted with the strongest negative importance in the detection of TN (green dots). Conversely, the more positive the PQ amplitudes in aVR, the greater the SHAP importance for detecting TP (red dots), which can be linked to the disturbed atrial depolarization in AF with a high probability for inversion of the f-/F-wave polarities. FP cases have positive P-waves with a PQa-mean box plot distribution quite similar to TP (median PQa-mean = 0.025 mV). FP can be associated with a variety of arrhythmias included in the non-AF group with disturbed polarity of atrial depolarization. FN cases are characterized by very small f-/F-wave amplitudes (median PQa-mean = 0 mV, interquartile range ±0.1 mV).
- PQa-mean (I, II): The typically positive P-wave amplitude in leads I and II for sinus rhythms is recognized by the green box plot distributions of TN (median PQa-mean = 0.12–0.15 mV). SHAP highlights the lower f-/F-amplitudes as the most important for TP (median PQa-mean = 0.06–0.08 mV). FP cases are non-AF cases with relatively low PQ-amplitudes (median PQa-mean = 0.09–0.1 mV), whereas FN cases are AF with enhanced high f-/F-wave amplitudes (median PQa-mean = 0.08–0.12 mV).
- PQi-std (V2, aVF, II): There is a clearly visible trend that a larger PQi-std is directly proportional to larger SHAP importance for TP (red dots), associated with a large variance of the PQ interval during the chaotic AV synchronization in AF. PQi variation is most prominent in lead V2 for TP (median PQi-std = 52 ms) compared with its limited value for TN (median PQi-std = 5 ms). FPs are non-AF rhythms with enhanced PQi-std (median PQi-std = 39 ms), whereas FNs are AF rhythms with relatively constant PQi, such as in some AFL (median PQi-std = 10 ms in V2).
- PQi-mean (aVR, aVL, I): The three leads present very similar distributions for TPs (median PQi-mean = 116 ms); however, different SHAP importance is given for low and high values of PQi-mean, i.e., lower PQi-mean values have higher TP importance for the lead aVR, whereas higher PQi-mean values have higher TP importance for leads aVL and I. This phenomenon is due to different PQi-mean distributions in the TN group, i.e., representing a longer PQ duration in the lead aVR (median PQi-mean = 160 ms), and shorter ones in leads aVL and I (median PQi-mean = 80 ms). This could be linked to different times of excitation of the right and left atria during sinus rhythm, shifting when the P-peak is detected in specific ECG leads, i.e., the earliest right atrium activation is detected in lead aVR, followed by the leftward and inferior direction of the activation detected in leads aVL and I. FP errors are non-AF arrhythmias with disturbed timing of the P-wave pattern, appearing with relatively equal PQ intervals in the three leads aVR, aVL, and I (median PQi-mean = 100–120 ms), which overlap with the f-/F-wave timing in AF. Although FN errors present a slightly shorter PQ interval than TP in lead aVL (median PQi-mean = 95 ms vs. 116 ms), such errors cannot be strongly linked to the disturbance of PQi-mean, because overlapping distributions for FN and TP groups are observed in leads aVR and I.
- PQa-std (V1, I, aVR): PQa-std is associated with a larger deviation of PQ amplitudes for TP and FP (median PQa-std = 0.04-0.05 mV) and lower deviations for TN and FN (median PQa-std = 0.01–0.03 mV), although the interquartile ranges overlap considerably between groups. This overlap is considered unreliable for AF detection by DenseNet, limiting the maximum SHAP global importance of PQa-std close to the 25% threshold (Figure 10a). Furthermore, the detailed analysis of the PQa-std (aVR) scatterplot in Figure 11 indicates that SHAP value importance is negative for TP, which means that this parameter biases the detection toward the non-AF class. Some explanations might account for the low PQ amplitudes and the difficulty in accurately measuring their small deviations.

**Figure 12:**
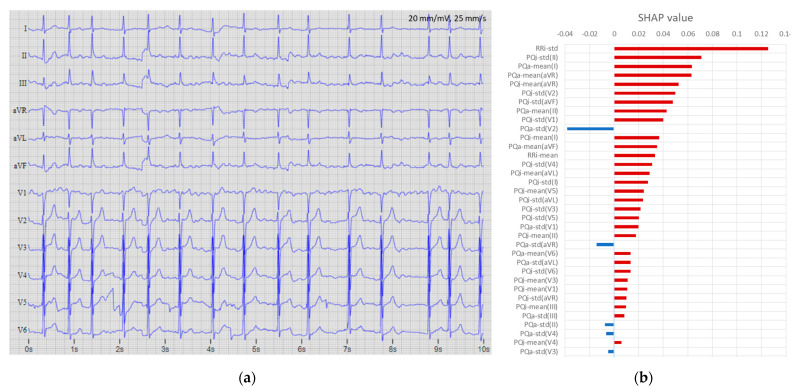
Example of a true positive case (file A6551 from database “CPSC2018 training set”): (**a**) 12-lead ECG (first 10 s of the record) with original annotation (AF); (**b**) top 35 features ranked by maximal absolute SHAP value of the DenseNet-12Leads [128,8,8] model, where red bars indicate the influence of the dominant number of features for the explicit detection of this case as AF with output probability of P_AF_ = 0.9999.

**Figure 13:**
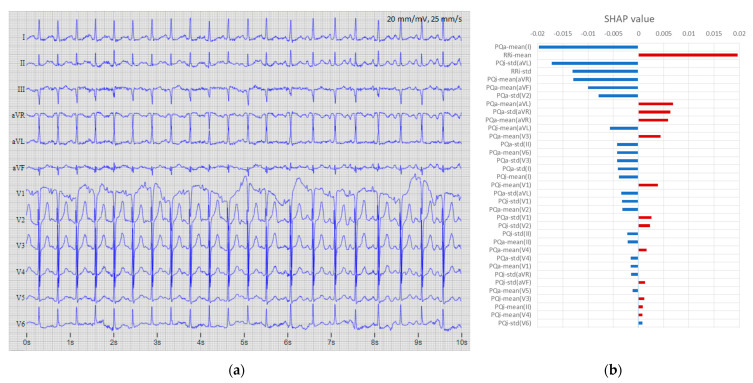
Example of a false negative error due to wrong AF annotation (file A6226 from database “CPSC2018 training set”): (**a**) 12-lead ECG (10 s total record duration) with original annotation (AF), which is inconsistent with the authors’ observations of a normal sinus rhythm with synchronized P-wave and QRS clearly seen in lead V1; (**b**) top 35 features ranked by maximal absolute SHAP value of the DenseNet-12Leads [128,8,8] model, where blue bars indicate the influence of the dominant number of features for the explicit detection of this case as non-AF with output probability of P_AF_ = 0.0114.

**Figure 14:**
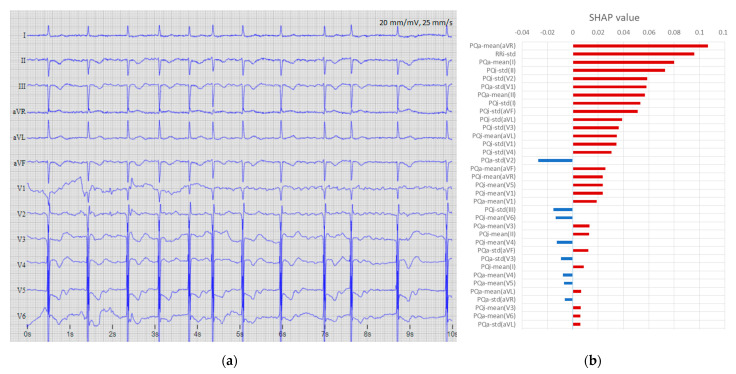
Example of a false positive error due to a wrong non-AF annotation (file Q3555 from “China 12-Lead ECG Challenge Database (CPSC2018-Extra)”): (**a**) 12-lead ECG (10 s total record duration) with original annotation for left bundle branch block, which is inconsistent with authors’ observation of an irregular AF rhythm with clearly visible f-waves in leads V1-V4; (**b**) top 35 features ranked by maximal absolute SHAP value of the DenseNet-12Leads [128,8,8] model, where red bars indicate the influence of the dominant number of features for the explicit detection of this case as AF with output probability of P_AF_ = 0.9999.

Figure 12, Figure 13 and Figure 14 show three examples of 12-lead ECGs and explanations of the respective features involved in the decisions of the DenseNet-12Lead [128,8,8] model. Figure 12 presents an AF record, which is correctly detected with a probability of P_AF_ > 0.99, supported by dominant features with a positive SHAP value in this case, maximal for RR-std, PQi-std(II, V2, aVF, V1), PQa-mean(I, aVR, II), and PQi-mean(aVR).

The next two examples provide insight into labeling inconsistencies and the reasons for erroneous performance of the model. Figure 13 presents a record that was annotated as AF and counted as a false negative error because DenseNet-12Lead detected non-AF rhythm. However, we suggest that this is a wrong AF annotation in a 12-lead ECG trace with normal sinus rhythm, confirmed by the P-waves and synchronized QRS complexes best discernible in lead V1. The probability of a definitive non-AF categorization (P_AF_ = 0.0114) is justified by the dominant number of features with negative SHAP values for this case, most notably including: PQa-mean (I, aVF), PQi-std(aVL), PQi-mean(aVR), and PQa-std(V2). The rhythm is relatively regular; therefore, the feature RRi-std is highly indicative of non-AF, but the rapid heart rate highlights RRi-mean as the single strongest indicator for AF.

The example in Figure 14 is annotated as non-AF and counted as a false positive error because DenseNet-12Lead detects AF rhythm with a high probability of P_AF_ > 0.99, supported by most features with positive SHAP values, most notably including: PQa-mean (aVR, I, II), RRi-std, PQi-std (II, V2, I, aVF), and PQa-std (V1). The authors’ observations of an irregular AF rhythm with clearly visible f-waves in leads V1-V4, however, suggest a wrong non-AF annotation.

## 4. Discussion

In view of prominent perspectives for the early and accurate detection of pathologic cardiac rhythms that focus on atrial fibrillation and atrial flutter, we suggest using AV synchronization in AF as an important diagnostic criterion when using one to twelve ECG leads. Standard diagnostic ECG features that represent the heart rate, lead-specific AV conduction time, and P-/f-wave amplitude (RR-interval, PQ-interval, and PQ amplitude, respectively) are calculated by simple rule-based methods, and their beat-to-beat mean values and standard deviations are interpreted by a dense neural network classifier. Although several neural architectures might show similar performance, optimization of the NN depth and width is an essential part of the design process. As shown in Figure 5, Figure 6, Figure 7 and Figure 8, some models have shown decreased validation performance either due to the randomness of the training runs, an insufficient number of hidden layers (i.e., all single-layer models), or an exhaustive number of neurons (related to the large number of training parameters). Therefore, applying grid search architectural optimization on the validation set is a required task, which led to the selection of the optimally trained DenseNet models with two or three dense layers. Their independent evaluation on the test set showed competitive performance with sensitivity and specificity values (Se, Sp) of: 87.9–88.3 and 90.5–91.5% for DenseNet [16,16,0] with primary leads (I or II), 90.7 and 94.2% for DenseNet [32,32,32] with six limb leads, 92.1 and 93.2% for DenseNet [32,32,4] with six chest leads, and 91.8 and 95.8% for DenseNet [128,8,8] with all 12 leads, although direct comparison with other studies is difficult due to different test sets, rhythm types, and performance metrics. To our knowledge, the Physionet CinC Challenge 2021 dataset has not been explored for binary AF/non-AF detection. Furthermore, due to the mixed annotations in some databases, we analyzed the mixed class of AFIB and AFL, although it is known that AFL is clinically recognized with limited atrial activity features and generally has more inaccurate detection. Future research should focus on the distinctive evaluation of AFIB and AFL in comparison with the vast taxonomy of cardiac arrhythmias in the large non-AF class of the Physionet CinC Challenge 2021 dataset.

Table 4 presents a comparative study of the performances of other AF detection methods found in the literature vs. our method. The disparities between the studies is shown in the detailed information on the applied methodology, test procedures, input lead sets, ECG databases, and evaluation accuracy metrics. The comparison should be interpreted with the following provisos:

- The few papers [62,79,80,81,82] reporting results in the Physionet/CinC Challenge 2021 database disclose accuracy metrics separately for AFIB and AFL, whereas Table 4 lists their average values.
- Even when the same datasets are used, direct comparison is not feasible due to the different test procedures—i.e., results are reported on either an independent test set (not used during training) or the validation dataset (total dataset or N-fold cross-validation) used in the NN training process.

The group of studies with single-lead ECG analysis have estimated their performance with several PhysioNet databases (MIT-BIH Atrial Fibrillation, Long-Term AF, MIT-BIH Arrhythmia) [22,23,24,28,30]. Their BAC is 7–11% points higher than that achieved in this study with one ECG lead (95.0–99.4 vs. 88.5%). We note that these databases contain a limited number of long-term Holter recordings and are not representative of the diversity of arrhythmias from the much larger number of patients available in the Physionet/CinC Challenge 2021 database. The effect of performance overestimation on uniform data might be evident in the fairly similar drop in performance (by about 10% points) when one of these methods [24] was cross-tested with the Physionet/CinC Challenge 2017 database (88.7 vs. 98.7%). These results, along with our cross-database test results in Table 3, strongly indicate that performance should be considered in the context of the test dataset used.

Among the numerous participants in the Physionet/CinC Challenge 2021, we were only able to compare those who reported F1 scores for AF detection in addition to the global Challenge score metric. The F1 scores of 12-lead models reported in [62,79] are comparable with this study (0.71, 0.72 vs. 0.77), whereas F1 in [80] is considerably lower (0.53 vs. 0.77). Furthermore, the F1 score of the two-lead model in [82] is comparable with the one-lead model in this study (0.53 vs. 0.55). The interpretation of the G metrics provided in [81] closely corresponds with the defined BAC metric, where this study is superior by 3% points for one-lead (85.4 vs. 88.0%), 6% points for 6-leads (86.7 vs. 92.5%), and 7% points for 12-leads (86.4 vs. 93.8%).

Another important issue is the clinical benefit of using diagnostic interpretive software, by general practitioners (GP) and practice nurses. Table 5 presents the disparity of results in two surveys for GP and practice nurse skills in AF interpretation [83,84], which demonstrate up to a 25% point difference in accuracy due to different human experience. In [83], 42 GPs and 41 practice nurses analyzed 2595 randomly selected ECGs from 25 GP practices and reported Se = 69–86%, Sp = 82–89% (manual interpretation) versus Se = 92%, Sp = 91% (manual plus automated interpretation). In [84], 457 GPs (out of 2239 who had been invited to participate in the study) analyzed 1613 single-lead ECG recordings and their reported accuracy in AF diagnosing was Se = 91.2%, Sp = 90.4% (GP manual interpretation) versus Se = 93.4%, Sp = 89.2% (GP interpretation after revision of the output of diagnostic interpretative software). Despite the high accuracy of GP manual ECG interpretation, 59% of those who responded to the questionnaire felt positively about the use of an automated algorithm in clinical practice. Moreover, 35% of GPs who refused to participate in the study but completed the questionnaire felt that their ECG reading skills were not sufficient to participate in such a study. Given this uncertainty in manual diagnosis, we suggest that the presented algorithm may be useful in supporting manual diagnosis, given its improved sensitivity by 2-12% points (GPs) and 15–19% points (nurses), and improved specificity by 2–11% points (GPs) and 6–10% points (nurses) in [83]. Moreover, our algorithm presented the same sensitivity and a 4.7% point improved specificity than the combination of GP and diagnostic interpretative software in [83]. 

This study justifies the importance of AV synchronization for AF prediction, using only three parameters (RR-interval, PQ-interval, and PQ-amplitude), whereas other state-of-the-art studies (Table 4) focus on complex feature maps (e.g., raw ECG data, Fourier spectrum, encoded-decoded heartbeats, short-term temporal ECG modulation features from scattering transforms, etc.) and many diagnostic criteria (e.g., classical interpretation methods). Such complex approaches are justified if many arrhythmias are to be classified, but the few parameters examined in this study were sufficient to detect AF rhythms. It is worth noting the advantage of implemented rule-based feature extraction methods, which have high computational efficiency in portable systems.

The focus of our study was on feature importance analysis, which was derived from SHAP evaluation of the decision-making process in the 12-lead DenseNet model, summarized as statistical distributions on the test set (Figure 10 and Figure 11), as well as a case overview of several ECG examples (Figure 12, Figure 13 and Figure 14). This analysis is important for understanding the strongest AF predictors found in the hidden layers of 12-lead DenseNet, as well as the causes of correct (TP and TN) and false detections (FN and FP), for a more comprehensive interpretation from a cardiologist’s point of view. The comparative estimation of 50 AV synchronization features in Figure 10a strongly indicates that ventricular synchronization (estimated as mean value and standard deviation of all beat-to-beat intervals in a record) is the most valuable indicator for detecting AF, with a relative importance in the range of 84–100%. Overall, atrial synchronization was noted to be much less important, with only twelve metrics reaching sensible levels of 25–60%, i.e., the mean PQ amplitude in leads (aVR, I, II), PQ interval deviation in leads (V2, aVF, II), mean PQ interval in leads (aVL, aVR, I), and mean PQ amplitude deviation in leads (V1, I, aVR). Based on the statistical graphs in Figure 11, these features can be comprehensively linked to common AF behavior such as rapid heart rate and enhanced heart rate variability, as well as the typical P-wave pattern behavior in sinus rhythm with the earliest negative P-peak deflection of the right atrium seen in the lead (aVR), positive P-peak deflection in leads (I, II), and late left atrium activation in lateral leads (aVL, I). Furthermore, the interquartile range of the PQ-interval deviation in V2 and inferior leads (aVF, II) was found to be prominently different between TN with synchronized atrial activation (1–20 ms) and TP with chaotic AV synchronization (30–60 ms). Although highlighted in leads (V1, I, aVR), the least important PQ characteristic was the mean PQ amplitude deviation, which showed considerable overlap between AF and non-AF rhythms. For this parameter, we accounted for the low PQ amplitudes and difficulty in accurately measuring their small deviations.

Although RR-intervals, PQ-intervals, and PQ amplitudes in 12-lead ECGs collectively contributed to a AF detection performance of 93.8%, there may be other AV synchronization features or ventricular polarization and depolarization features that could additionally improve diagnostic accuracy, e.g., reducing FP among the variety of non-AF arrhythmias that present disturbed AV synchronization. This is an area for future research.

## 5. Conclusions

Despite the very distinctive AF behavior of atrial and ventricular irregularities, the automated detection of these rhythms remains a challenging task, considering the potential paroxysmal occurrences of AF and possibility of coexistence with other arrhythmias in high-risk patients. In view of the significant burden associated with AF complications, for both patients and healthcare systems, early AF diagnosis is of crucial importance. Therefore, attempts to detect AF should be extended beyond the clinical setting to long-term ECG monitoring techniques that can identify even brief and asymptomatic AF episodes, as well as preventive examinations being carried out by GPs and practice nurses instead of only expert cardiologists.

In view of prominent perspectives for early and accurate AF detection, the major contributions of this study are:

- A few comprehensive measures of AV synchronization, related to the mean and standard deviation of the heart rate, AV conduction time, and P-/f-wave amplitude (RR-interval, PQ-interval, and PQ amplitude, respectively) in 12-lead ECGs were shown to be feasible for AF detection.
- Advanced NN classifiers with one to three hidden dense layers and up to 128 neurons per layer were optimized to detect AF with input features from one, six, and twelve ECG leads.
- Performance generalizability was demonstrated using independent datasets for training (50,332 records), validation (14,235 records), and test (6978 records), part of the six largest PhysioNet CinC Challenge 2021 databases, which were rich in data from healthy controls and patients showing various arrhythmias, including AF.
- We elucidated the decision-making process of the DenseNet model by the SHAP method and highlighted the 14 most important AF predictors. Statistical analysis of their distributions comprehensively explained the causes of correct (TP, TN) and false detections (FN, FP).

Although performance was limited to the AF diagnostic potential of the few studied AV synchronization features, these features would be easily measured using portable ECG devices, and would correctly alert for AF in 87.6% (single-lead model) to 91.8% (12-lead model) at the cost of 11.5% and 4.2% false positive alarms, respectively. Such early warning would certainly reduce the complications of AF, which typically remains untreated for a long time.

Other clinical benefits include the SHAP value importance, which can be used in combination with a human expert’s ECG interpretation to change the focus of the expert eye from broad observation of 12-lead ECG morphology to only a few AV synchronization findings that are strongly predictive for AF or non-AF arrhythmias. Their diagnostic value may outperform GP and practice nurse AF diagnoses by (2–19%) and may reduce false AF alarms by (2–11%), according to reports of some clinical surveys (Table 5). The deduced results of this study are representative of AV synchronization findings across a broad taxonomy of cardiac arrhythmias in large 12-lead ECG databases.

In conclusion, given that AF is the most common arrhythmia, has a major impact on morbidity and mortality, and can manifest asymptomatically in physically active patients, its automatic screening is of high priority. In this regard, the proposed AF detection technique based on a neural network classifier and analysis of a physiologically reasoned and intuitive input feature set has the potential of a screening utility or decision-support application in clinical practice. The direct clinical impact of such a novel technology will require further investigation in prospective clinical studies.

## Figures and Tables

**Table 1:** Number of selected 12-lead ECG records from six CinC Challenge 2021 databases, allocated randomly and patient-wise into subsets for training (70%), validation (20%), and test (10%). The class imbalance rate of AF (8%) vs. non-AF (92%) was approximately preserved in the different subsets.

Databases	ECG Records (Total Number)	ECG Duration (s)	Training		Validation		Test	
			AF	Non-AF	AF	Non-AF	AF	Non-AF
CPSC2018 training set	5074	6–144	711	2866	203	795	102	397
China 12-Lead ECG Challenge database (CPSC2018-Extra)	1233	6–144	115	785	33	190	17	93
PTB-XL electrocardiography database	20,113	10–120	378	15,119	108	3461	53	994
Georgia 12-Lead ECG Challenge database	8565	5–10	130	6579	38	1245	18	555
Chapman-Shaoxing database	8244	10	650	5171	186	1568	93	576
Ningbo database	28,316	10	1929	15,899	551	5857	276	3804
Total	71,545	5–144	3913	46,419	1119	13,116	559	6419

**Table 2:** Validation and test performance of the optimal DenseNet models for binary detection of AF/non-AF rhythm.

Architecture	Lead Set	Number of Input Features	Validation Dataset				Test Dataset			
			BAC (%)	Se (%)	Sp (%)	F1 Score	BAC (%)	Se (%)	Sp (%)	F1 Score
DenseNet-SingleLeads [16,16,0]	I	6	-	-	-	-	89.3	86.2	92.3	0.629
	II	6	-	-	-	-	88.5	84.3	92.8	0.63
	III	6	-	-	-	-	86.2	82.7	89.8	0.551
	aVR	6	-	-	-	-	84.5	81.9	87.1	0.497
	aVL	6	-	-	-	-	85.9	83.7	88.1	0.523
	aVF	6	-	-	-	-	88.5	85.0	92.0	0.615
	V1	6	-	-	-	-	87.2	82.8	91.6	0.593
	V2	6	-	-	-	-	89.4	88.0	90.7	0.598
	V3	6	-	-	-	-	88.9	87.8	89.9	0.577
	V4	6	-	-	-	-	88.6	87.8	89.3	0.565
	V5	6	-	-	-	-	88.4	87.5	89.4	0.565
	V6	6	-	-	-	-	88.4	84.8	91.9	0.612
DenseNet-SingleLeads [16,16,0]	Total single leads	6	91.1	92.6	89.5	0.587	88.0	87.6	88.5	0.547
DenseNet-LimbLeads [32,32,32]	Limb leads	26	94.7	94.6	94.9	0.744	92.4	90.7	94.2	0.704
DenseNet-ChestLeads [32,32,4]	Chest leads	26	94.6	95.2	94.0	0.716	92.7	92.1	93.2	0.683
DenseNet-12Leads [128,8,8]	12-leads	50	94.9	93.3	96.6	0.800	93.8	91.8	95.8	0.767

The symbol (-) indicates that validation performance was not calculated for specific single leads because total single leads were evaluated as part of the global validation dataset during the training process.

**Table 3:** Detailed performance of binary AF/non-AF rhythm detection across six test datasets, reported for the complete 12-lead ECG feature set (DenseNet-12Leads [128,8,8] model).

Test Datasets	TP	FN	TN	FP	BAC (%)	Se (%)	Sp (%)	F1 Score
CPSC2018 training set	100	2	351	46	93.2	98.0	88.4	0.806
China 12-Lead ECG Challenge database	15	2	88	5	91.4	88.2	94.6	0.811
PTB-XL electrocardiography database	50	3	965	29	95.7	94.3	97.1	0.758
Georgia 12-Lead ECG Challenge database	12	6	531	24	81.2	66.7	95.7	0.444
Chapman-Shaoxing database	88	5	551	25	95.1	94.6	95.7	0.854
Ningbo database	248	28	3667	137	93.1	89.9	96.4	0.750
Total	513	46	6153	266	93.8	91.8	95.9	0.767

**Table 4:** Comparison with other published studies presenting machine-learning AF detection algorithms. The performance metrics are the same as reported in original articles.

Study	Methodology	Database	Se (%)	Sp (%)	BAC (%)	F1 score
	Single-lead ECG analysis					
This study	Features: 6 AV synchronization features Classifier: DenseNet-SingleLeads [16,16,0] Test: Independent dataset	PhysioNet/CinC Challenge 2021	87.6	88.5	88.0	0.547
[81]	Features: 109 heart rate variability features Classifier: NN Test: 3-fold cross validation	PhysioNet/CinC Challenge 2021 *	NA	NA	G = 85.4 ^#,$^	NA
[24] ^†^	Features: Deep heartbeat encoding and aggregation by RNN Classifier: Fully connected NN Test: 10-fold cross-validation	Physionet/CinC Challenge 2017 MIT-BIH AF MIT-BIH Arrhythmia	79.9 98.7 96.1	97.5 98.6 98.9	88.7 98.7 97.5	0.81 0.99 0.93
[22]	Features: RR intervals and atrial activity Classifier: Decision rules Test: Long-Term AF database	MIT-BIH AF (training) Long-Term AF (test)	97.4 95.6	98.7 99.3	98.1 97.5	NA NA
[23]	Features: QT interval and heart rate Classifier: Error-Correcting Output Codes Test: 10-fold cross-validation	MIT-BIH AF MIT-BIH Arrhythmia	100	90.0	95.0	NA
[28] ^&,†^	Features: Raw ECG Classifier: CNN and Elman NN Test: 10-fold cross-validation	MIT-BIH AF MIT-BIH Arrhythmia	99.6 98.9	99.1 98.6	99.4 98.8	NA NA
[30]	Features: Raw ECG Classifier: 13-layer CNN Test: Independent dataset	Long-Term AF, Paroxysmal AF, AF termination challenge, Fantasia, MIT-BIH Arrhythmia, Indonesian database	93.7	96.9	95.3	NA
	**Two-lead ECG analysis**					
[82]	Features: Encoder-decoder of raw ECG Classifier: NN Test: 3-fold cross validation	PhysioNet/CinC Challenge 2021 ^*^	NA	NA	NA	0.53 ^$^
	**Six lead-ECG analysis**					
This study	Features: 26 AV synchronization features Classifier: DenseNet-LimbLeads [32,32,32] Test: Independent dataset	PhysioNet/CinC Challenge 2021	90.7	94.2	92.4	0.704
	Features: 26 AV synchronization features Classifier: DenseNet-ChestLeads [32,32,4] Test: Independent dataset	PhysioNet/CinC Challenge 2021	92.1	93.2	92.7	0.683
[81]	Features: 109 heart rate variability features Classifier: NN Test: 3-fold cross validation	PhysioNet/CinC Challenge 2021 ^*^	NA	NA	G = 86.7 ^#,$^	NA
	**12-lead ECG analysis**					
This study	Features: 50 AV synchronization features Classifier: DenseNet-12Leads [128,8,8] Test: Independent dataset	PhysioNet/CinC Challenge 2021	91.8	95.8	93.8	0.767
[62]	Features: Raw ECG and Fourier spectrum Classifier: 2 CNN, 1 fully connected layer Test: Validation data used during training	PhysioNet/CinC Challenge 2021 ^*^	NA	NA	NA	0.71 ^$^
[79]	Features: Short-term temporal ECG modulation features from scattering transform Classifier: CNN and LSTM layers Test: 10-fold cross-validation	PhysioNet/CinC Challenge 2021 ^*^	NA	NA	NA	0.72 ^$^
[80]	Features: Raw ECG Classifier: InceptionTime CNN Test: Validation data used during training	PhysioNet/CinC Challenge 2021 ^*^	52.0 ^$^	NA	NA	0.53 ^$^
[81]	Features: 109 heart rate variability features Classifier: NN Test: 3-fold cross validation	PhysioNet/CinC Challenge 2021 ^*^	NA	NA	G = 86.4 ^#,$^	NA

* PhysioNet/CinC Challenge 2021 includes databases: CPSC, PTB, PTB-XL, INCART, Chapman-Shaoxing, Ningbo, and Georgia databases. ^#^ The values are for the G metric (𝐺 = 𝑠qrt(𝑆𝑒∗𝑆𝑝)) as presented in the original paper. ^$^ The accuracy metrics in the original papers are presented separately for AFIB and AFL. Here, we used an average value. ^&^ The non-AF signals in this study are only normal sinus rhythms. ^†^ These studies present performances of more than one AF detection methodology. Here, the best accuracy results are used for comparison.

**Table 5:** Comparison with other published studies on GP and practice nurse skills in AF interpretation. The performance metrics are the same as reported in original articles.

Lead Set	Study	Method	Se	Sp
1-lead	[83]	42 GPs	85.5	86.4
		41 practice nurses	68.7	82.7
	[84]	457 GPs		
		GP manual interpretation	91.2	90.4
		GP + Diagnostic interpretative software *	93.4	89.2
	This study	6 AV synchronization features DenseNet-SingleLeads [16,16,0]	87.6	88.5
Limb-leads	[83]	42 GPs	82.5	88.5
		41 practice nurses	72.0	83.4
	This study	26 AV synchronization features DenseNet-LimbLeads [32,32,32]	90.7	94.2
Chest-leads	[83]	42 GPs	84.8	86.4
		41 practice nurses	68.7	82.8
	This study	26 AV synchronization features DenseNet-ChestLeads [32,32,4]	92.1	93.2
12-leads	[83]	42 GPs	79.8	91.6
		41 practice nurses	77.1	85.1
		Diagnostic interpretative software	83.3	99.1
		GP + Diagnostic interpretative software **	91.9	91.1
	This study	50 AV synchronization features DenseNet-12Leads [128,8,8]	91.8	95.8

* The GP decides whether AF is present or not. ** AF positive decision is taken if the GP, software, or both classify the rhythm as AF.

## Data Availability

Publicly available datasets were analyzed in this study. This data can be found in the PhysioNet databank: https://physionet.org/content/challenge-2021/1.0.2/, last accessed on 26 June 2022.

## References

[1] Shah S.R., Luu S.W., Calestino M., David J., Christopher B. (2018). Management of atrial fibrillation-flutter: Uptodate guideline paper on the current evidence. J. Community Hosp. Intern. Med. Perspect..

[2] Naccarelli G., Varker H., Lin J., Schulman K. (2009). Increasing prevalence of atrial fibrillation and flutter in the United States. Am. J. Cardiol..

[3] Naydenov S., Runev N., Manov E., Vasileva D., Rangelov Y., Naydenova N. (2018). Risk Factors, co-morbidities and treatment of in-hospital patients with atrial fibrillation in Bulgaria. Medicina.

[4] Naydenov S., Runev N., Manov E. (2021). Are Three Weeks of Oral Anticoagulation Sufficient for Safe Cardioversion in Atrial Fibrillation?. Medicina.

[5] January C.T., Wann L.S., Alpert J.S., Calkins H., Cigarroa J.E., Cleveland J.C., Conti J.B., Ellinor P.T., Ezekowitz M.D., Field M.E. (2014). 2014 AHA/ACC/HRS guideline for the management of patients with atrial fibrillation: A report of the American College of Cardiology/American Heart Association Task Force on Practice Guidelines and the Heart Rhythm Society. J. Am. Coll. Cardiol..

[6] Gertsch M. (2004). The Normal ECG and its (Normal) Variants. The ECG: A Two-Step Approach to Diagnosis.

[7] Hindricks G., Potpara T., Dagres N., Arbelo E., Bax J., Blomstrom-Lundqvist C. (2020). 2020 ESC Guidelines for the diagnosis and management of atrial fibrillation developed in collaboration with the European Association of Cardio-Thoracic Surgery (EACTS). Eur. Heart J..

[8] Dotsinsky I. (2007). Atrial wave detection algorithm for discovery of some rhythm abnormalities. Physiol. Meas..

[9] Du X., Rao N., Qian M., Liu D., Li J., Feng W., Yin L., Chen X. (2014). A novel method for real-time atrial fibrillation detection in electrocardiograms using multiple parameters. Ann. Noninvasive Electrocardiol..

[10] Christov I., Bortolan G., Daskalov I. Sequential Analysis for Automatic Detection of Atrial Fibrillation and Flutter. Proceedings of the 2001 Computers in Cardiology Conference.

[11] Ladavich S., Ghoraani B. (2015). Rate-independent detection of atrial fibrillation by statistical modeling of atrial activity. Biomed. Signal Processing Control.

[12] Linker D. (2016). Accurate, Automated Detection of Atrial Fibrillation in Ambulatory Recordings. Cardiovasc. Eng. Technol..

[13] Petrėnas A., Marozas V., Sörnmo L. (2015). Low-complexity detection of atrial fibrillation in continuous long-term monitoring. Comput. Biol. Med..

[14] Lake D., Moorman J. (2011). Accurate estimation of entropy in very short physiological time series: The problem of atrial fibrillation detection in implanted ventricular devices. Am. J. Physiol. Heart Circ. Physiol..

[15] Andersen R.S., Peimankar A., Puthusserypady S. (2019). A deep learning approach for real-time detection of atrial fibrillation. Expert Syst. Appl..

[16] Ebrahimzadeh E., Kalantari M., Joulani M., Shahraki R.S., Fayaz F., Ahmadi F. (2018). Prediction of paroxysmal Atrial Fibrillation: A machine learning based approach using combined feature vector and mixture of expert classification on HRV signal. Comput. Methods Programs Biomed..

[17] Boon K.H., Khalil-Hani M., Malarvili M.B., Sia C.W. (2016). Paroxysmal atrial fibrillation prediction method with shorter HRV sequences. Comput. Methods Programs Biomed..

[18] Faust O., Shenfield A., Kareem M., San T.R., Fujita H., Acharya U.R. (2018). Automated detection of atrial fibrillation using long short-term memory network with RR interval signals. Comput. Biol. Med..

[19] Babaeizadeh S., Gregg R., Helfenbein E., Lindauer J., Zhou S. (2009). Improvements in atrial fibrillation detection for real-time monitoring. J. Electrocardiol..

[20] Petrėnas A., Sörnmo L., Lukoševicius A., Marozas V. (2015). Detection of occult paroxysmal atrial fibrillation. Med. Biol. Eng. Comput..

[21] Larburu N., Lopetegi T., Romero I. (2011). Comparative study of algorithms for atrial fibrillation detection. Comput. Cardiol..

[22] Tuboly G., Kozmann G., Kiss O., Merkely B. (2021). Atrial fibrillation detection with and without atrial activity analysis using lead-I mobile ECG technology. Biomed. Signal Processing Control.

[23] Soni E., Nagpal A., Chopra K. (2022). Atrial fibrillation discrimination for real-time ECG monitoring based on QT interval variation. Indian J. Sci. Technol..

[24] Dubatovka A., Buhmann J.M. (2022). Automatic detection of atrial fibrillation from single-lead ECG using deep learning of the cardiac cycle. BME Front..

[25] Xia Y., Wulan N., Wang K., Zhang H. (2018). Detecting atrial fibrillation by deep convolutional neural networks. Comput. Biol. Med..

[26] Rouhi R., Clausel M., Oster J., Lauer F. (2021). An interpretable hand-crafted feature-based model for atrial fibrillation detection. Front. Physiol..

[27] Krasteva V., Christov I., Naydenov S., Stoyanov T., Jekova I. (2021). Application of dense neural networks for detection of atrial fibrillation and ranking of augmented ECG feature set. Sensors.

[28] Wang J. (2020). Automated detection of atrial fibrillation and atrial flutter in ECG signals based on convolutional and improved Elman neural network. Knowl.-Based Syst..

[29] Baek Y.S., Lee S.C., Choi W., Kim D.H. (2021). A new deep learning algorithm of 12-lead electrocardiogram for identifying atrial fibrillation during sinus rhythm. Sci. Rep..

[30] Tutuko B., Nurmaini S., Tondas A.E., Rachmatullah M.N., Darmawahyuni A., Esafri R., Firdaus F., Sapitri A.I. (2021). AFibNet: An implementation of atrial fibrillation detection with convolutional neural network. BMC Med. Inform. Decis. Mak..

[31] Fan X., Yao Q., Cai Y., Miao F., Sun F., Li Y. (2018). Multiscaled fusion of deep convolutional neural networks for screening atrial fibrillation from single lead short ECG recordings. IEEE J. Biomed. Health Inform..

[32] Attia Z.I., Noseworthy P.A., Lopez-Jimenez F., Asirvatham S.J., Deshmukh A.J., Gersh B.J., Carter R.E., Yao X., Rabinstein A.A., Erickson B.J. (2019). An artificial intelligence-enabled ECG algorithm for the identification of patients with atrial fibrillation during sinus rhythm: A retrospective analysis of outcome prediction. Lancet.

[33] Fujita H., Cimr D. (2019). Computer aided detection for fibrillations and flutters using deep convolutional neural network. Inf. Sci..

[34] Zhao Z., Särkkä S., Rad A.B. (2020). Kalman-based spectro-temporal ECG analysis using deep convolutional networks for atrial fibrillation detection. J. Signal Process. Syst..

[35] Nurmaini S., Tondas A.E., Darmawahyuni A., Rachmatullah M.N., Partan R.U., Firdaus F., Tutuko B., Pratiwi F., Juliano A.H., Khoirani R. (2020). Robust detection of atrial fibrillation from short-term electrocardiogram using convolutional neural networks. Future Gener. Comput..

[36] Baalman S.W., Schroevers F.E., Oakley A.J., Brouwer T.F., van der Stuijt W., Bleijendaal H., Ramos L.A., Lopes R.R., Marquering H.A., Knops R.E. (2020). A morphology based deep learning model for atrial fibrillation detection using single cycle electrocardiographic samples. Int. J. Cardiol..

[37] Mousavi S., Afghah F., Acharya U.R. (2020). HAN-ECG: An interpretable atrial fibrillation detection model using hierarchical attention networks. Comput. Biol. Med..

[38] Cao P., Li X., Mao K., Lu F., Ning G., Fang L., Pan Q. (2020). A novel data augmentation method to enhance deep neural networks for detection of atrial fibrillation. Biomed. Signal Process. Control.

[39] Petmezas G., Haris K., Stefanopoulos L., Kilintzis V., Tzavelis A., Rogers J.A., Katsaggelos A.K., Maglaveras N. (2021). Automated atrial fibrillation detection using a hybrid CNN-LSTM network on imbalanced ECG datasets. Biomed. Signal Process. Control.

[40] Zhang X., Li J., Cai Z., Zhang L., Chen Z., Liu C. (2021). Over-fitting suppression training strategies for deep learning-based atrial fibrillation detection. Med. Biol. Eng. Comput..

[41] Tran L., Li Y., Nocera L., Shahabi C., Xiong L. (2020). MultiFusionNet: Atrial fibrillation detection with deep neural networks. AMIA Jt. Summits Transl. Sci. Proc..

[42] Jin Y., Qin C., Huang Y., Zhao W., Liu C. (2020). Multi-domain modeling of atrial fibrillation detection with twin attentional convolutional long short-term memory neural networks. Knowl.-Based Syst..

[43] Fan X., Hu Z., Wang R., Yin L., Li Y., Cai Y. (2020). A novel hybrid network of fusing rhythmic and morphological features for atrial fibrillation detection on mobile ECG signals. Neural Comput. Appl..

[44] Lai D., Bu Y., Su Y., Zhang X., Ma C.S. (2020). Non-standardized patch-based ECG lead together with deep learning based algorithm for automatic screening of atrial fibrillation. IEEE J. Biomed. Health Inform..

[45] Sawhney N.S., Anousheh R., Chen W.C., Feld G.K. (2009). Diagnosis and management of typical atrial flutter. Cardiol. Clin..

[46] Boyer M., Koplan B. (2005). Atrial Flutter. Circulation.

[47] Daoud E.G., Morady F. (1998). Pathophysiology of Atrial Flutter. Annu. Rev. Med..

[48] Morrison S., Macfarlane P.W. (2000). Computer Detection of Atrial Flutter. Ann. Noninvasive Electrocardiol..

[49] National Institute for Health and Care Excellence (NICE) (2021). Atrial Fibrillation: Diagnosis and Management. NICE Guideline [NG196]. https://www.nice.org.uk/guidance/ng196.

[50] Zink M.D., Laureanti R., Hermans B.J.M., Pison L., Verheule S., Philippens S., Pluymaekers N., Vroomen M., Hermans A., van Hunnik A. (2022). Extended ECG improves classification of paroxysmal and persistent atrial fibrillation based on P- and f-waves. Front. Physiol..

[51] Sasaki N., Okumura Y., Watanabe I., Madry A., Hamano Y., Nikaido M., Nagashima K., Sonoda K., Kogawa R., Takahashi K. (2015). Frequency analysis of atrial fibrillation from the specific ECG leads V7–V9: A lower DF in lead V9 is a marker of potential atrial remodeling. J. Cardiol..

[52] Steinberg J.S., O’Connell H., Li S., Ziegler P.D. (2018). Thirty-second gold standard definition of atrial fibrillation and its relationship with subsequent arrhythmia patterns: Analysis of a large prospective device database. Circ. Arrhythmia Electrophysiol..

[53] Ballatore A., Matta M., Saglietto A., Desalvo P., Bocchino P., Gaita F., De Ferrari G.M., Anselmino M. (2019). Subclinical and Asymptomatic Atrial Fibrillation: Current Evidence and Unsolved Questions in Clinical Practice. Medicina.

[54] Rosero S.Z., Kutyifa V., Olshansky B., Zareba W. (2013). Ambulatory ECG monitoring in atrial fibrillation management. Prog. Cardiovasc. Dis..

[55] Duarte R., Stainthorpe A., Mahon J., Greenhalgh J., Richardson M., Nevitt S., Kotas E., Boland A., Thom H., Marshall T. (2019). Lead-I ECG for detecting atrial fibrillation in patients attending primary care with an irregular pulse using single-time point testing: A systematic review and economic evaluation. PLoS ONE.

[56] Reyna M., Sadr N., Alday E., Gu A., Shah A., Robichaux C., Bahrami Rad A., Elola A., Seyedi S., Ansari S. Will Two Do? Varying Dimensions in Electrocardiography: The PhysioNet/Computing in Cardiology Challenge 2021. Proceedings of the 2021 Computing in Cardiology Conference (CinC 2021).

[57] Kropf M., Hayn D., Morris D., Radhakrishnan A.K., Belyavskiy E., Frydas A., Pieske-Kraigher E., Pieske B., Schreier G. (2018). Cardiac anomaly detection based on time and frequency domain features using tree-based classifiers. Physiol. Meas..

[58] Shao M., Bin G., Wu S., Bin G., Huang J., Zhou Z. (2018). Detection of atrial fibrillation from ECG recordings using decision tree ensemble with multi-level features. Physiol. Meas..

[59] Sodmann P., Vollmer M., Nath N., Kaderali L. (2018). A convolutional neural network for ECG annotation as the basis for classification of cardiac rhythms. Physiol. Meas..

[60] Rizwan M., Whitaker B., Anderson D. (2018). AF detection from ECG recordings using feature selection, sparse coding, and ensemble learning. Physiol. Meas..

[61] Mukherjeez A., Choudhuryz A., Datta S., Puri C., Banerjee R., Singh R., Ukil A., Bandyopadhyay S., Pal A., Khandelwal S. (2019). Detection of atrial fibrillation and other abnormal rhythms from ECG using a multi-layer classifier architecture. Physiol. Meas..

[62] Wickramasinghe N.L., Athif M. (2022). Multi-label classification of reduced-lead ECGs using an interpretable deep convolutional neural network. Physiol. Meas..

[63] Goldberger A.L., Amaral L.A.N., Glass L., Hausdorff J.M., Ivanov P.C., Mark R.G., Mietus J.E., Moody G.B., Peng C.-K., Stanley H.E. (2000). PhysioBank, PhysioToolkit, and PhysioNet: Components of a new research resource for complex physiologic signals. Circulation.

[64] Reyna M., Sadr N., Gu A., Perez Alday E.A., Liu C., Seyedi S., Shah A., Clifford G.D. (2021). Will Two Do? Varying Dimensions in Electrocardiography: The PhysioNet/Computing in Cardiology Challenge 2021 (version 1.0.2). Comput. Cardiol..

[65] Xie C., McCullum L., Johnson A., Pollard T., Gow B., Moody B. (2021). Waveform Database Software Package (WFDB) for Python (version 3.4.1). Circulation.

[66] Levkov C., Mihov G., Ivanov R., Daskalov I., Christov I., Dotsinsky I. (2005). Removal of power-line interference from the ECG: A review of the subtraction procedure. BioMed. Eng. OnLine.

[67] Bortolan G., Christov I., Simova I., Dotsinsky I. (2015). Noise processing in exercise ECG stress test for the analysis and the clinical characterization of QRS and T wave alternans. Biomed. Signal Process. Control.

[68] Christov I., Neycheva T., Schmid R., Stoyanov T., Abächerli R. (2017). Pseudo real-time low-pass filter in ECG, self-adjustable to the frequency spectra of the waves. Med. Biol. Eng. Comput..

[69] Christov I.I. (2004). Real time electrocardiogram QRS detection using combined adaptive threshold. BioMed. Eng. Online.

[70] Daskalov I.K., Christov I.I. (1999). Electrocardiogram signal preprocessing for automatic detection of QRS boundaries. Med. Eng. Phys..

[71] Christov I., Simova I. (2006). Fully automated method for QT interval measurement in ECG. Comput. Cardiol..

[72] Christov I., Krasteva V., Simova I., Neycheva T., Schmid R. (2018). Ranking of the most reliable beat morphology and heart rate variability features for detection of atrial fibrillation in short single lead ECG. Physiol. Meas..

[73] Malik M. (1996). Heart rate variability. Standards of measurement, physiological interpretation, and clinical use. Task Force of the European Society of Cardiology and the North American Society of Pacing and Electrophysiology. Eur. Heart J..

[74] Ioffe S., Szegedy C. Batch normalization: Accelerating deep network training by reducing internal covariate shift. Proceedings of the 32nd International Conference on Machine Learning (ICML 2015).

[75] Ramsundar B., Zadeh R.B., Roumeliotis R., Young A. (2018). Chapter 4. Fully Connected Deep Networks. TensorFlow for Deep Learning: From Linear Regression to Reinforcement Learning.

[76] Jekova I., Krasteva V. (2021). Optimization of end-to-end convolutional neural networks for analysis of out-of-hospital cardiac arrest rhythms during cardiopulmonary resuscitation. Sensors.

[77] Shapley L.S. (1953). A value for n-person games. Contrib. Theory Games.

[78] Molnar C. (2022). Interpretable Machine Learning. A Guide for Making Black Box Models Explainable.

[79] Warrick P.A., Lostanlen V., Eickenberg M., Homsi M.N., Rodrıguez A.C., Anden J. Arrhythmia classification of reduced-lead electrocardiograms by scattering-recurrent networks. Proceedings of the 2021 Computing in Cardiology Conference (CinC 2021).

[80] Crocker H.J., Costall A.W. An inception time-inspired convolutional neural network to detect cardiac abnormalities in reduced-lead ECG data. Proceedings of the 2021 Computing in Cardiology Conference (CinC 2021).

[81] Jiménez-Serrano S., Rodrigo M., Calvo C.J., Castells F., Millet J. Multiple cardiac disease detection from minimal-lead ECG combining feedforward neural networks with a one-vs-rest approach. Proceedings of the 2021 Computing in Cardiology Conference (CinC 2021).

[82] Rodrigues R., Couto P. Semi-supervised learning for ECG classification. Proceedings of the 2021 Computing in Cardiology Conference (CinC 2021).

[83] Mant J., Fitzmaurice D.A., Hobbs F.D., Jowett S., Murray E.T., Holder R., Davies M., Lip G. (2007). Accuracy of diagnosing atrial fibrillation on electrocardiogram by primary care practitioners and interpretative diagnostic software: Analysis of data from screening for atrial fibrillation in the elderly (SAFE) trial. BMJ.

[84] Karregat E., Himmelreich J., Lucassen W., Busschers W., van Weert H., Harskamp R. (2021). Evaluation of general practitioners’ single-lead electrocardiogram interpretation skills: A casevignette study. Fam. Pract..

